# Robustly interrogating machine learning-based scoring functions: what are they learning?

**DOI:** 10.1093/bioinformatics/btaf040

**Published:** 2025-01-28

**Authors:** Guy Durant, Fergus Boyles, Kristian Birchall, Brian Marsden, Charlotte M Deane

**Affiliations:** Department of Statistics, University of Oxford, St Giles', Oxford OX1 3LB, United Kingdom; Department of Statistics, University of Oxford, St Giles', Oxford OX1 3LB, United Kingdom; LifeArc, Stevenage SG1 2FX, United Kingdom; Nuffield Department of Medicine, University of Oxford, Oxford OX3 7BN, United Kingdom; Department of Statistics, University of Oxford, St Giles', Oxford OX1 3LB, United Kingdom

## Abstract

**Motivation:**

Machine learning-based scoring functions (MLBSFs) have been found to exhibit inconsistent performance on different benchmarks and be prone to learning dataset bias. For the field to develop MLBSFs that learn a generalizable understanding of physics, a more rigorous understanding of how they perform is required.

**Results:**

In this work, we compared the performance of a diverse set of popular MLBSFs (RFScore, SIGN, OnionNet-2, Pafnucy, and PointVS) to our proposed baseline models that can only learn dataset biases on a range of benchmarks. We found that these baseline models were competitive in accuracy to these MLBSFs in almost all proposed benchmarks, indicating these models only learn dataset biases. Our tests and provided platform, ToolBoxSF, will enable researchers to robustly interrogate MLBSF performance and determine the effect of dataset biases on their predictions.

**Availability and implementation:**

https://github.com/guydurant/toolboxsf.

## 1 Introduction

Predicting the binding affinity of a protein–ligand complex from its 3D structure has been extensively researched in the past decade (Meli *et al.* 2022). However, doing so accurately and for any protein–ligand complex still poses a significant challenge in computational chemistry (Mobley and Gilson 2017). Accurately predicting binding affinity would aid in structure-based drug discovery, where the chemical structure of a drug is designed based on the structure of its target, as it would allow design hypotheses to be tested in silico. One proposed methodology, scoring functions (Goodsell *et al.* 1996), which estimate binding affinity based on the features of a single protein–ligand complex structure, offer fast predictions and are suited for high throughput hit identification and lead optimization (Bissantz *et al.* 2000).

Docking software, such as AutoDock 4 (Morris *et al.* 2009), AutoDock Vina (Trott and Olson 2010), GOLD (Verdonk *et al.* 2003), and Glide (Friesner *et al.* 2004) commonly use scoring functions to predict the structure of the bound ligand (the pose), its binding affinity and its rank compared to other proposed poses. These scoring functions use either molecular force fields (Huang *et al.* 2006), statistical potentials (Gohlke *et al.* 2000), or linear combinations of empirical terms (Krammer *et al.* 2005). Advancements in machine learning (ML) have enabled the development of ML-based scoring functions (MLBSFs) that outperform these other scoring functions in accuracy for predicting binding affinity. Initially, these scoring functions used classical ML techniques, e.g. tree-based models, and simple features extracted from the protein–ligand complex structure (Ballester and Mitchell 2010, Durrant and McCammon 2011, Zilian and Sotriffer 2013, Ballester *et al.* 2014, Li *et al.* 2015, Wang and Zhang 2017, Meli *et al.* 2021).

With the emergence of deep learning techniques, scoring functions based on the convolutional neural network (CNN) architecture to predict the binding affinity only were built and trained on explicit, voxelised representations of the ligand-protein complex (Francoeur *et al.* 2020) [e.g. Pafnucy (Stepniewska-Dziubinska *et al.* 2018) and KDeep (Jiménez *et al.* 2018)]. Newer deep learning methods such as graph neural networks (GNNs) represented atoms as nodes, bonds as edges and used message-passing to pass feature vectors across the graphs to learn higher representations for predicting binding affinity (Karlov *et al.* 2020, Li *et al.* 2021, Moon *et al.* 2022, Volkov *et al.* 2022, Scantlebury *et al.* 2023). Despite the plethora of methods published, there is no clear consensus on which architecture should be used and how to improve scoring function accuracy for predicting binding affinity, given the small differences in performance observed between the methods on the standard benchmarks (Carlson *et al.* 2016, Su *et al.* 2019).

Most MLBSFs are trained on the PDBBind database (Wang *et al.* 2004), which consists of thousands of protein–ligand complex crystal structures with binding affinity data extracted from the literature. Complexes in CASF 2016, the most popular benchmark for scoring function performance, have very high similarity to data points within the standard training dataset (PDBBind) resulting in an over-optimistic measurement of accuracy as MLBSFs can memorize data similarity or ‘bias’ instead of relevant biophysics (Scantlebury *et al.* 2023). This has also been a problem in the adjacent virtual screening field for classifying binders and nonbinders (Wallach and Heifets 2018, Sieg *et al.* 2019). Alternative methods of interrogation have been proposed by us and others, these include clustered cross-validation (Zhu *et al.* 2022), leave-cluster-out cross-validation (Kramer and Gedeck 2010), time-splits (Volkov *et al.* 2022), and removing training data similar to the test data (Boyles *et al.* 2020, Scantlebury *et al.* 2023). Unfortunately, due to the widespread use of the CASF 2016 benchmark for evaluating models, researchers can only compare their proposed model to others using that benchmark, exacerbating the problem of inadequate scoring function evaluation. Furthermore, MLBSFs are benchmarked and tested on accurate crystal structures but often will be used for scoring predicted docked ligand poses against noncognate or predicted structures in a real-world drug discovery setting. These noisy structures are likely to be less accurately predicted compared to the crystal structure, yet this impact has been explored in a limited manner for a few scoring functions by us and others (Francoeur *et al.* 2020, McNutt *et al.* 2021, Shen *et al.* 2021, Boyles *et al.* 2022, Wong *et al.* 2022, Scardino *et al.* 2023). We and others have demonstrated that models trained only on ligand and/or protein identities without explicitly including the interactions between them perform surprisingly well on CASF 2016 (Boyles *et al.* 2020, Volkov *et al.* 2022). It can be difficult to definitively prove that models are learning bias in the dataset due to the ‘black box’ nature of many ML models. Learning bias is not inherently unhelpful and can be useful if models are used within the domain they have been trained. Prospective success is also possible with MLBSFs, whether they have learnt bias or not (Hu *et al.* 2022).

Here, we present a platform for interrogating scoring function performance, called ToolBoxSF. We explored the ability of these models to predict binding affinity values, not to classify binders and nonbinders. First, we reimplemented a diverse set of MLBSFs: RFScore (Ballester and Mitchell 2010), Pafnucy (Stepniewska-Dziubinska *et al.* 2018), PointVS (Scantlebury *et al.* 2023), SIGN (Li *et al.* 2021), and OnionNet-2 (Wang *et al.* 2021b), to use a consistent API and provide new tests and baseline models to interrogate their performance. We found that simple baseline models trained on only ‘dataset biases’ had competitive performance to the tested scoring functions in accuracy on a range of benchmarks. We also found behaviours of these MLBSFs that also suggest they are exploiting these dataset biases. The provided platform and results should enable researchers to fully and robustly interrogate their models and determine the effect of dataset biases on their predictions.

## 2 Materials and methods

### 2.1 Training dataset

For consistency, we trained all models on PDBBind 2020 General, the most recent release at the time of writing (Liu *et al.* 2014). It consists of crystal structures of bound protein–ligand complexes with an associated binding affinity label (*K_i_*, *K_D_*, or *IC*_50_). We excluded complexes that could not be processed by the latest version of RDKit (2023.03.01) (Landrum 2023) or by OpenBabel (3.1.1) (O’Boyle *et al.* 2011). This left 19 079 complexes for training and testing. Structures were prepared as described below (Docking) except the ligand coordinates were not recalculated. In this work, IC_50_, K_*i*_, and K_*D*_ were treated as the same, a common approach in the field (Meli *et al.* 2022) despite the values not being strictly interchangeable (Kalliokoski *et al.* 2013). The pK for each compound was calculated by the following equation:


(1) $$pK=-{\;\text{log}\;}_{10}(K_{i}\;\text{or}\;K_{d}{\;\text{or}\;\text{IC}}_{50})$$

### 2.2 Docking

Docking was done using Smina, a fork of AutoDock Vina (Koes *et al.* 2013). The default parameters were chosen except ‘exhaustiveness’ (set to 12) and ‘autobox_add’ (set to 8 Å). The protonation of the ligand and protein were kept consistent with those provided by PDBBind. The MOL2 ligand files provided in PDBBind General 2020 were converted into SDF format for consistency with the docked poses. Their 3D coordinates were recalculated using the ETKDG method from RDKit (Riniker and Landrum 2015) before docking to ensure the docking software was not able to use the crystal pose to influence its conformational search. Protein files had water molecules and any other nonchain atoms removed.

### 2.3 Benchmark preparation

To generate a benchmark where ligand bias cannot be used for accurate predictions, the 0 Ligand Bias benchmark, we clustered identical molecules that were bound to different proteins by matching their InChI-Key (Pletnev *et al.* 2012) and took clusters whose mean pK value was within 6 and 7 pK units and whose variance was larger than 1 pK unit. This left 365 complexes as a test set. These two final steps were done to remove identical ligands that had highly similar pK values and to ensure that predicting the mean of the clusters did not artificially increase the accuracy. For example, if two clusters had values concentrated around a low pK and a high pK value respectively, predicting the mean of each cluster would result in a high correlation between the predicted and true pK values. Peptides, defined as any entry in PDBBind with a ligand code with the letters ‘MER’, were held out to create the Peptides Holdout (2574 complexes). This benchmark tested the scoring functions’ ability to score peptides having never been exposed to them in the training dataset, to be accurate on this benchmark a scoring function must learn an understanding of biophysics that generalizes from smaller molecules to peptides. For the 2019 Holdout set, as done in Volkov *et al.* (2022), we took any PDBBind data point with a crystal structure produced from 2019 or later as a test set (1511 complexes). This time split was designed to create a tougher test for scoring functions compared to CASF 2016. To determine the effect of protein structure accuracy on performance, we redocked (Redocked) and crossdocked CASF 2016 ligands into protein conformations that were either bound but with high pocket similarity [CrossDocked (Best)], low pocket similarity [CrossDocked (Worst)], into apo structures (Apo), predicted AlphaFold 2 structures (Alphafold 2), and a random wrong protein (Wrong Protein). The details of how the conformations were picked can be found in the Supplementary Information (S.I.) (S1).

To generate a diverse range of docking errors, we redocked the ligand back into the cognate structure of each protein–ligand complex of CASF 2016, 2019 Holdout and 0 Ligand Bias. We increased the ‘autobox_add’ parameter to 20 Å and ‘num_modes’ to 1000 for Smina. To generate more poses close in accuracy to the true pose, we also minimized the crystal pose using the ‘minimize’ option. Poses were binned by RMSD to the crystal pose using the following ranges: 0–1, 1–2, 2–4, 4–6, 6–8, 8–10, 10–15, 15–20, 20–25 and 25–30Å. If available, the pose closest to the mean of the bin was chosen for each test set. To explore the impact of clashes on the models, the crystal pose of the ligand for each complex from CASF 2016, 2019 Holdout and 0 Ligand Bias was progressively translated into the protein 1 Å at a time, ten times. To calculate a normal vector for this translation, we normalized the direction vector between the closest ligand and closest protein atom in each protein–ligand complex.

### 2.4 Implementation of scoring functions and models

To compare performance across a range of scoring functions, five popular and diverse models were selected from the literature: RFScore (Ballester and Mitchell 2010), PointVS (Scantlebury *et al.* 2023), Pafnucy (Stepniewska-Dziubinska *et al.* 2018), SIGN (Li *et al.* 2021), and OnionNet-2 (Wang *et al.* 2021b). RFScore was one of the first methods to use ML to predict binding affinity, it uses Random Forest models and counts of protein and ligand elements that are within 12 Å of each other. Pafnucy uses a CNN architecture and 3D voxelized representations of the protein–ligand complex. OnionNet-2 also uses a CNN with a 2D image of the counts of each specific amino acid-ligand atom interaction with differing thresholded distances. SIGN and PointVS both use GNNs with attention layers for the edges. PointVS is also pre-trained to classify pose accuracy within 2 Å and uses this as a prior for its prediction of binding affinity. All differences between the original implementations and our modified implementations can be found in the S.I. (S2.1).

We developed four separate baseline models that represent models that can only learn ;bias’ in the dataset. All were developed using tree-based models with architecture and hyperparameters chosen by the FLAML package (Wang *et al.* 2021a) using 5-fold cross-validation of the training dataset with CASF 2016 excluded. The LigandBias model is based on the simple QSAR-like model from Boyles *et al.* (2020). However, unlike QSAR methods it is applicable to any protein and not a single protein like a standard QSAR model. Where 1D and 2D descriptors from the RDKit package were calculated to featurise only the ligand. Any descriptor that produced NaN values or extremely large values was excluded, leaving 195 features. The LigandBias model will always predict the same value of affinity for a ligand no matter what protein it binds to as it cannot see the ligand. Therefore, it can only memorize ligand identity and its performance can be ascribed to learning the ligand bias from the data. The ProteinBias model used counts of each amino acid within the pocket as a feature vector. We defined the protein pocket as any amino acid that had an atom within 15 Å of any ligand atom. The impact of this threshold on model performance is explored in the S.I. (S2.3). These features give the ProteinBias model the identity of the amino acids but not proximity to each other or ligand atoms, and so severely limits the structural information in the features, and so can only memorize pocket identity or bias. We ensembled LigandBias and ProteinBias predictions to give the EnsembleBias model, which is unable to see both biases at once. Our final model, the BothBias model concatenates features from both the ProteinBias and LigandBias models, which can learn from both sets of bias at once but is still prevented from learning from the 3D structure, and so learning biophysics. The full information on algorithms and hyperparameters for each baseline model is provided in the S.I. (S2.2). We also scored all the test sets using Smina (Koes *et al.* 2013) as a baseline for the performance of a non-MLBSF.

The data produced in this work and code for these models have been developed into an easy-to-use platform, called ToolBoxSF, to robustly compare to proposed models from the community and examine if they are learning more than bias. All models have been installed into separate Singularity containers to allow instant and easy use of the models for training or predictions. These wrappers are available on GitHub and as pre-built Singularity containers (https://github.com/guydurant/toolboxsf).

### 2.5 Metrics

Scoring function accuracy was calculated between their predicted and true values using bootstrapped Pearson’s *R*, *R*^2^ and root mean squared error (RMSE) values where data points were sampled with replacement 10 000 times to produce 95% confidence intervals. Accuracy measured using Pearson’s *R* is presented in the main text as is the most commonly used metric in this field, results for the other two metrics are provided in the S.I. (S3–S11).

## 3 Results

### 3.1 Existing benchmarks

Evaluation of scoring function accuracy has typically been done using the CASF 2016 benchmark, so we first benchmarked each model on this set for Pearson’s *R*, the commonly used metric used to assess scoring function accuracy (Table 1, CASF 2016), with results for *R*^2^ and RMSE in the S.I. (S3.1). However, the similarity between training (PDBBind) and test set (CASF 2016) makes this an unsuitable benchmark for assessing MLBSF generalizability (Scantlebury *et al.* 2023). We compared five different models that featurise the protein–ligand complex differently: RFScore (Ballester and Mitchell 2010), Pafnucy (Stepniewska-Dziubinska *et al.* 2018), PointVS (Scantlebury *et al.* 2023), SIGN (Li *et al.* 2021), and OnionNet-2 (Wang *et al.* 2021b). We retrained these scoring functions on our training sets and compared their performance against our baseline models which are unable to learn anything about the structure or interactions of the protein–ligand complex. The model trained on both protein and ligand features that contain no 3D information and so only dataset biases (‘BothBias’) had the highest values, although RFScore, SIGN and OnionNet-2 were within confidence intervals for all three metrics demonstrating that learning biophysics from 3D information is not necessary for close to state-of-the-art performance on the standard CASF 2016 benchmark (Wang *et al.* 2021b). High performance on this benchmark has been shown to not be indicative of generalizability (Volkov *et al.* 2022, Zhu *et al.* 2022, Scantlebury *et al.* 2023) but our result goes one step further and demonstrates that even attempting to learn biophysics from structures of the protein–ligand complex provides no additional accuracy. Volkov *et al.* (2022) in an attempt to account for this bias, proposed a time-split where PDBBind data points from 2019 and later were held out as a test set (Table 1, 2019 Holdout). BothBias baseline is within confidence intervals for all metrics with OnionNet-2, the highest performing. This outcome indicates that a time-based split may not be suitable for demonstrating that a scoring function has learnt concepts of biophysics instead of dataset bias. Although both of these benchmarks have value in evaluating the accuracy of scoring functions, it is clear that other benchmarks are required to determine whether a model would be capable of generalizing to novel protein or ligand families.

**Table 1.** Pearson’s *R* between predicted and true pK values for protein–ligand complexes for our baseline models (LigandBias, ProteinBias, EnsembleBias, and BothBias), a non-MLBSF (Smina), and five commonly used MLBSFs (RFScore, PointVS, Pafnucy, SIGN, and OnionNet-2) on four benchmark datasets (CASF 2016, 2019 Holdout, Peptides Holdout, and 0 Ligand Bias).^a^

Method	CASF 2016	2019 Holdout	Peptides Holdout	0 Ligand Bias
LigandBias	0.76 ± 0.06	0.59 ± 0.03	0.23 ± 0.04	0.08 ± 0.11
ProteinBias	0.75 ± 0.07	0.59 ± 0.04	0.32 ± 0.04	**0.41 ± 0.10**
EnsembleBias	0.82 ± 0.04	0.68 ± 0.03	**0.37 ± 0.04**	0.27 ± 0.11
BothBias	**0.85 ± 0.03**	0.67 ± 0.03	0.35 ± 0.04	0.27 ± 0.12
Smina	0.59 ± 0.08	0.36 ± 0.04	0.19 ± 0.04	0.12 ± 0.10
RFScore	0.82 ± 0.04	0.64 ± 0.03	0.33 ± 0.04	0.24 ± 0.10
PointVS	0.79 ± 0.04	0.66 ± 0.03	**0.37 ± 0.04**	0.28 ± 0.10
Pafnucy	0.74 ± 0.06	0.60 ± 0.04	**0.37 ± 0.04**	0.17 ± 0.11
SIGN	0.82 ± 0.04	0.66 ± 0.03	0 .34 ± 0.04	0.27 ± 0.10
OnionNet-2	0.82 ± 0.04	**0.70 ± 0.03**	0.36 ± 0.04	0.35 ± 0.10

a See methods for further details of scoring functions and dataset creation. The highest values are in bold and underlined, with any value within the highest values’ confidence intervals underlined. Error ranges represent the 95% confidence intervals from bootstrapped Pearson’s *R* (*N* = 10 000).

### 3.2 New proposed benchmarks

We propose two benchmarks which evaluate the generalizability of ML scoring functions in different ways. The first utilizes the difference between the properties of peptide-protein complexes and ligand-protein complexes found within PDBBind 2020. We removed any peptide-containing complex as a hold-out set from the training dataset. Peptides are difficult to score due to their inherent flexibility and are often much larger than the other ligands in PDBBind (London *et al.* 2010). We also explored the impact of restricting peptides to more ‘drug-like’ lengths in the S.I. (S3.2). This makes it a difficult benchmark but success would demonstrate that the models have learnt an understanding of biophysics, such as entropy and changes in solvation, that generalizes to peptides. The results in Table 1 show that the BothBias had performance within confidence intervals of the highest performing methods for Pearson’s R. We also note that ProteinBias performed the most accurately in *R*^2^ and RMSE, demonstrating the need for analysis of scoring function accuracy using more than one metric. Our second benchmark takes advantage of scoring functions tending to learn ligand-specific bias in that they are poor at differentiating between the same ligand bound to different proteins (Boyles *et al.* 2020). We identified identical ligands within PDBBind 2020 General that had existed two or more times in the dataset and filtered to ensure these identical ligands’ mean and variance of pKs were centred but spread across the mean pK of the PDBBind dataset (i.e. the training dataset). These groups of identical ligands were then combined into a single set as the 0 Ligand Bias set. On this test set, ProteinBias had the highest performance, with OnionNet-2 and PointVS within confidence intervals for some of the metrics. Notably, BothBias performed worse. This demonstrates that ignoring the ligand is sufficient for the highest performance currently on this test set. Protein bias is useful due to the similarity of protein pockets between the test and train sets [88% of test set pockets have the same Pfam ID as pockets in the train set (Finn *et al.* 2014)]. Low performance across all models tested, across all metrics, indicates that this is a challenging benchmark. Furthermore, the incredibly similar performance of BothBias and EnsembleBias models in all benchmarks indicates that BothBias is not constraining interactions using the 2D features, as it is impossible for EnsembleBias to do so. In the rest of this paper, we refer to only the results of BothBias for brevity. These benchmarks demonstrate that current scoring functions are not able to significantly outperform models trained on bias. Therefore these MLSBFs are both learning bias that does not generalize to this test set and learning little or nothing further.

### 3.3 Effect of protein structure accuracy on performance

**Figure 1.**
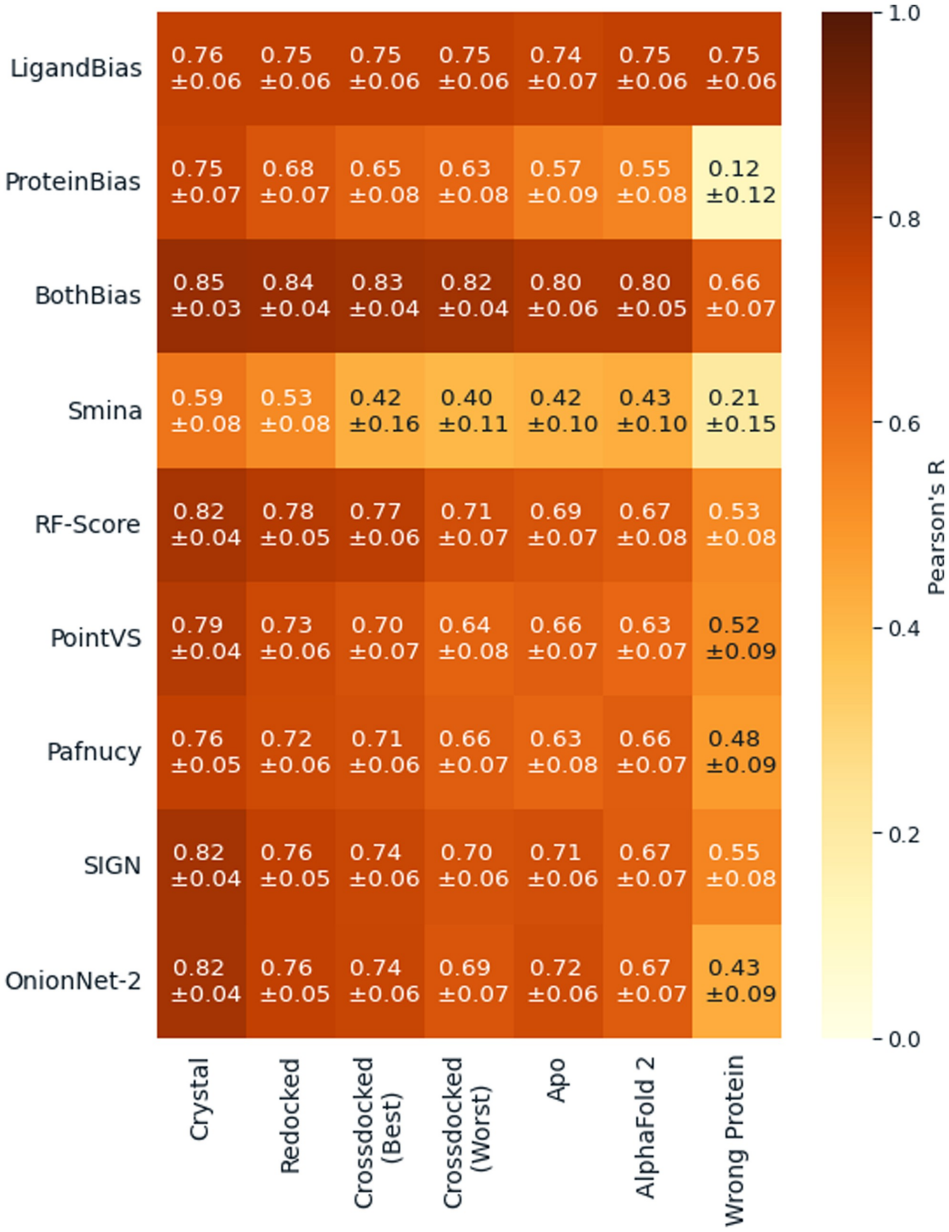
Pearson’s *R* between predicted and true pK values for protein–ligand complexes for our baseline models (LigandBias, ProteinBias, and BothBias), a non-MLBSF (Smina), and five commonly used MLBSFs (RFScore, PointVS, Pafnucy, SIGN, and OnionNet-2) on alternate CASF 2016 complex type test sets. Errors are the 95% confidence intervals from the bootstrapped Pearson’s *R* (*N* = 10 000). Results using *R*^2^ and RMSE are provided in the S.I. (S5).

One deficiency in using the test sets used above as benchmarks or held-out tests is that they only measure accuracy for scoring crystal structures. Typically scoring functions are used to score docked poses against crystal or predicted structures that might not have an accurate active site conformation for the docked ligand. This introduces noise into the structure as docking predictions may not find the specific interactions or recapture the true binding pose of the crystal structure. To explore the impact of this noise on accuracy, we created alternate docked versions of the CASF 2016 benchmark, which is made up of five structures, each bound to a different ligand, for each of 57 types of proteins (so 285 complexes total) and so contains alternate conformations for the same protein to dock into. We produced six test sets where we re-docked the ligand back into the cognate protein structure (Fig. 1, Redocked), cross-docked it into a conformation most similar to its own [Fig. 1, Crossdocked (Best)] and again into a conformation most dissimilar [Fig. 1, Crossdocked (Worst)]. We also docked the ligand into apo (unbound) structures (Fig. 1, Apo), predicted AF2 structures (Fig. 1, Alphafold 2), and a random protein from CASF 2016 not from its family as a baseline (Fig. 1, Wrong Pocket). The differences in structure are shown for a case study (PDB:1E66) in the S.I. (S1.3) to display both the change in 3D structure and how this affects which interactions are formed.

These increasingly noisy types of structure demonstrated decreased accuracy when scored by all scoring functions, as shown in Fig. 1. The scoring functions were able to maintain a correlation with the true values even if the ligand was docked into a completely different protein demonstrating a lower bound of accuracy caused by predictions being dominated by identifying the ligand rather than the nature of the complex. The BothBias model does not appear to be affected as much by the increasing noise as its ligand features are not impacted by changes in conformation and the number of amino acids in the protein pocket does not change significantly across the complex types. These results also suggest as expected that measuring performance on crystal structures provides an upper limit of the ability of scoring functions that is unlikely to be replicated if used in a drug discovery campaign (Brown *et al.* 2009).

### 3.4 Effect of docking accuracy on performance

**Figure 2.**
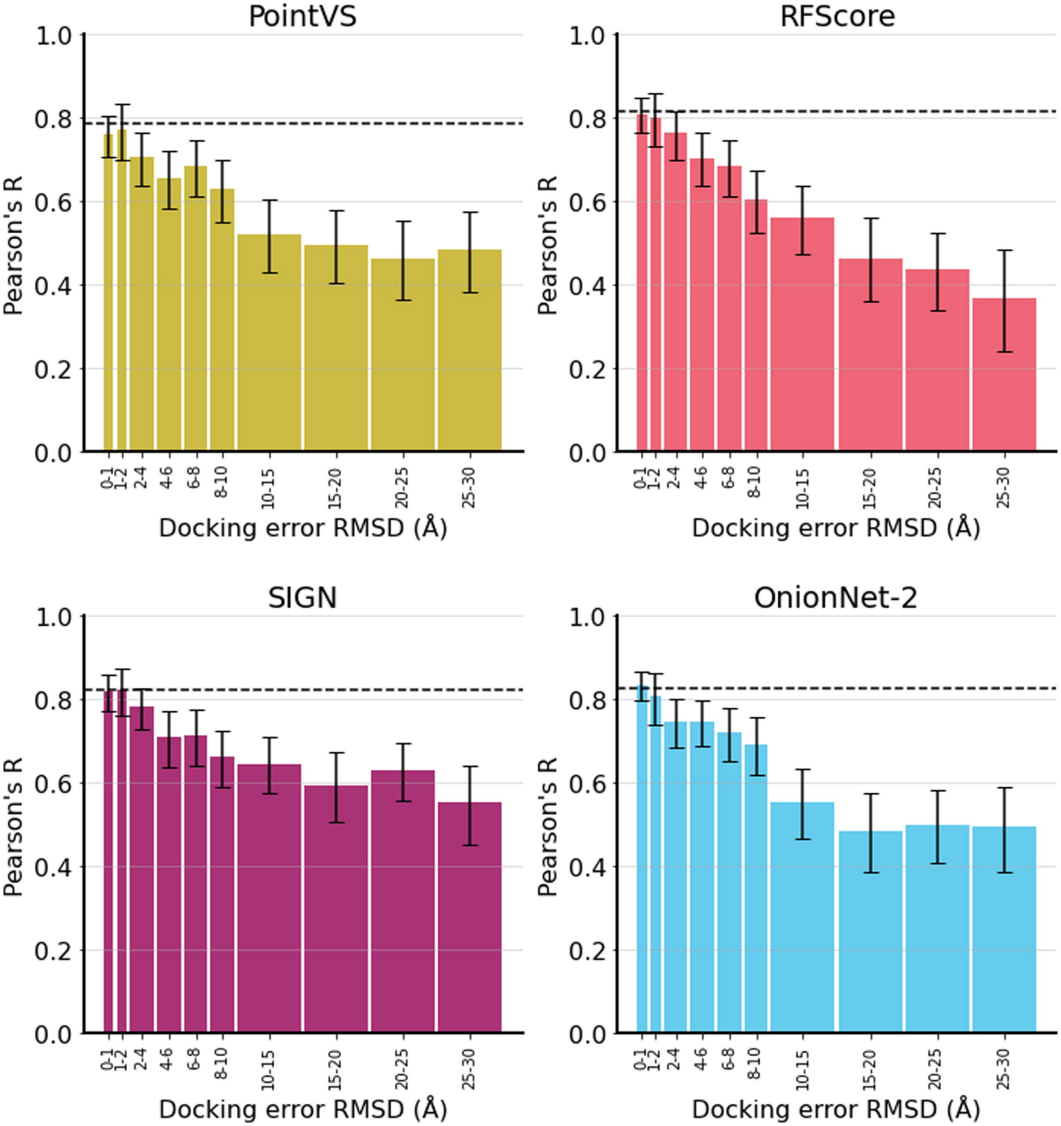
Pearson’s *R* between predicted and true pK values for protein–ligand complexes for four selected MLBSFs, PointVS, RFScore, SIGN, and OnionNet-2, on different accuracy poses of CASF 2016 complexes. Accuracy on the crystal structures of CASF 2016 is shown as a dashed black line. Errors are the 95% confidence intervals from the bootstrapped Pearson’s *R* (*N* = 10 000). Results using *R*^2^ and RMSE are provided in the S.I. (S6).

To measure the impact of docking accuracy, we considered a diverse set of poses for the CASF 2016 complexes, binned by RMSD. We tested all scoring functions and baseline models but here highlight the results of PointVS, RFScore, SIGN, and OnionNet-2 (Fig. 2). The results for all other methods can be found in the S.I. (S6). When high-accuracy poses were used, models retained high predictive accuracy relative to scoring the crystal structures when scored by different scoring functions.

However, as docking error increased, correlation with true values decreased and ultimately plateaued at 10 Å, except for RFScore which continued to decline beyond this point. This plateauing occurs even for Smina, probably due to its ligand-size bias (Chang *et al.* 2010). Similar to the complex type tests, there was a lower bound for this decrease in performance even at extreme docking errors (25–30Å), where the ligand is no longer bound in the correct site, showing again the models were relying on ligand bias to score protein–ligand complexes. We also explored this effect on 2019 Holdout and 0 Ligand Bias complexes and found the same trend (S.I.) (S7, S8). This demonstrates that although docking accuracy is important for binding affinity prediction accuracy, bias is currently a more significant driver of scoring performance as there is still correlation with true values for highly inaccurate poses.

### 3.5 Clashes

**Figure 3.**
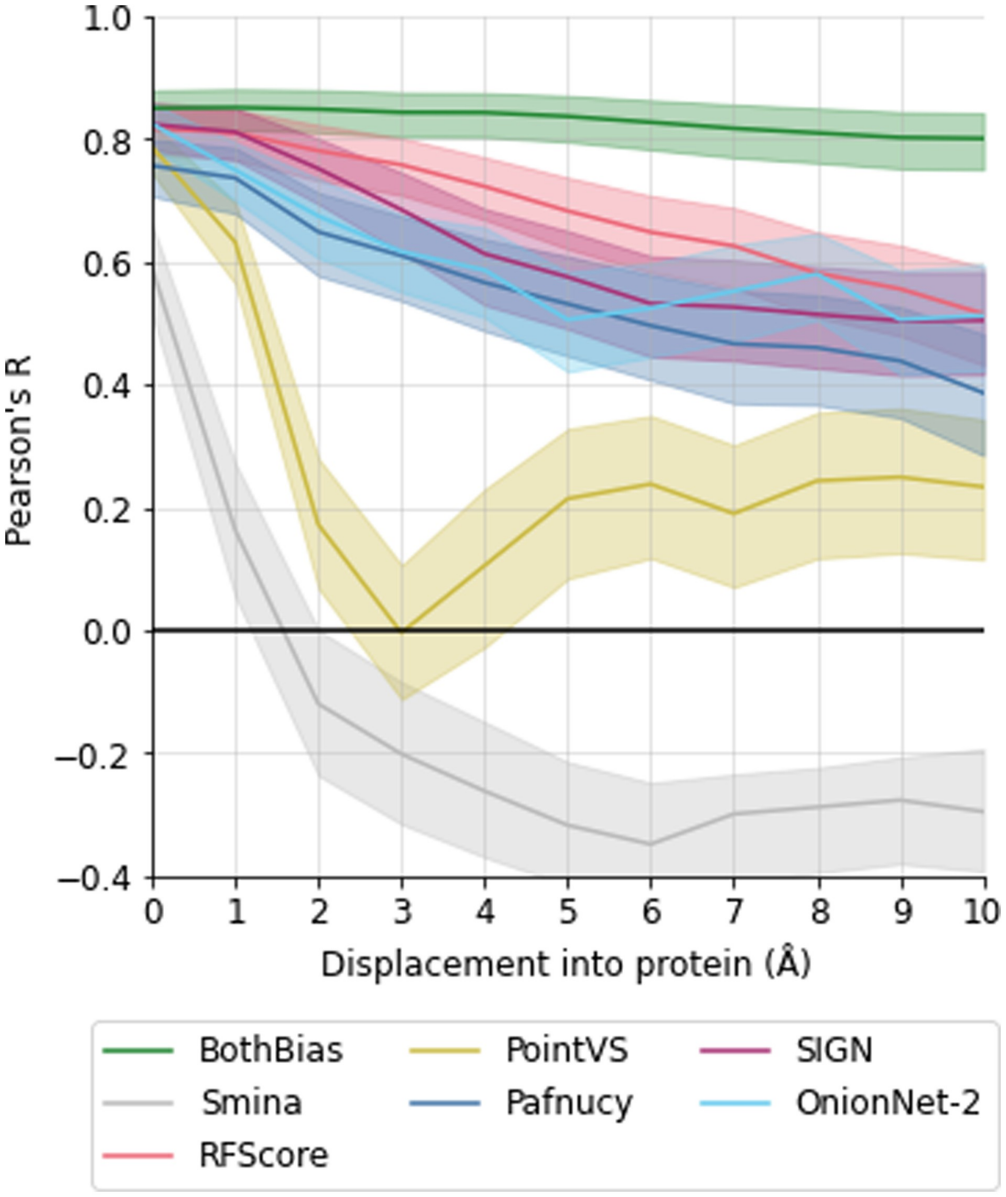
Pearson’s *R* between predicted and true pK values for protein–ligand complexes for one baseline model (BothBias), a non-MLBSF (Smina), and five commonly used MLBSFs (RFScore, PointVS, Pafnucy, SIGN, and OnionNet-2) on progressively displaced ligands into the protein originally from CASF 2016 crystal structures. Errors are the 95% confidence intervals from the bootstrapped Pearson’s *R* (*N* = 10 000). Results using *R*^2^ and RMSE are provided in the S.I. (S9).

Finally, we investigated scoring function performance when there were clashes in the protein–ligand complex by creating a series of structures where the ligand was translated into the protein for each CASF 2016 complex. Although it is unlikely these scoring functions will come across these types of structures in a drug discovery scenario, the overlap of ligand and protein structure provides such an unrealistic structure with many clashes and few interactions between the protein and ligand. Therefore it is expected that the scoring function should fail to accurately predict binding affinity. The MLBSFs displayed greater sensitivity to translation than the BothBias baseline model; however, most scoring functions displayed only a gradually decreasing performance as the clashes became increasingly severe, again indicating a lower bound (Fig. 3). This indicates that the scoring functions only recognize that the ligand is further from the binding site, rather than detecting the unphysical clashes with the protein.

The exceptions to these trends are Smina and PointVS, which are both co-trained or pre-trained for pose prediction and demonstrate higher sensitivity to clashes with low or no accuracy on complexes with significant clashes. Again, we also explored this effect on 2019 Holdout and 0 Ligand Bias complexes and found the same trends as for CASF 2016 (S10, S11). This suggests that considering pose quality in the training process provides scoring functions with the ability to discriminate between clashing, overlapping structures, and true protein–ligand complex structures.

## 4 Conclusion

In this work, we have demonstrated that state-of-the-art performance on CASF 2016 can be achieved by baseline models using only protein and ligand bias. We propose the 0 Ligand Bias and Peptide Holdout test sets which either explicitly penalize learning ligand bias or require a greater understanding of biophysics, as tougher benchmarks and novel thresholds for improvement from the field. Five popular MLBSFs were equalled or outperformed by baseline models in our tests, indicating that the performance of these scoring functions may be the result of learning dataset bias. We believe our baseline models offer a yardstick for the field as if any proposed scoring function can outperform them, they will have learned more than simple dataset bias.

We examined the effect of noise in the 3D structure of the protein–ligand complex on scoring function performance. The noise introduced by using inaccurate active site conformations or docked poses both resulted in degradation of accuracy in relationship to the amount of noise. This noise being either how dissimilar the active sites are to the cognate crystal structure or the RMSD difference of the pose to the crystal pose. However, both decreases in correlation to true values had a lower bound showing indifference to the 3D structure input and instead relying on recognizing the identity of the ligand.

A further proof that these models are not necessarily learning relevant biophysics is their insensitivity to serious steric clashes between protein and ligands. Translation of the crystal pose into the surface of the protein resulted in a gradual decrease in performance indicating that the scoring functions were only able to recognize that the ligand was further from its true location. The exceptions to this trend, PointVS, and Smina, were either pre-trained or developed for pose classification or ranking respectively. These exceptions suggest that scoring functions trained to predict only binding affinity do not learn how sensible a pose is, whilst co-training for another task, such as pose classification, forces it to appreciate clashes. However, it must be noted that Smina never outperformed any of these MLSBSFs in accuracy on any benchmark.

Overall, this work has provided a meta-analysis of scoring functions and created baseline models that equal existing scoring function accuracy and has provided train-test splits that can help identify if proposed models have learnt more than this simple dataset bias. For the field to progress it will be necessary to design and train models in such a way that they cannot achieve apparent success on benchmarks simply by learning dataset biases. For researchers to prove their proposed scoring functions have learnt more than dataset bias, we have presented rigorous tests and baseline models that can be used for comparisons. All code and dataset splits can be accessed here: (https://github.com/guydurant/toolboxsf).

## Supplementary Material

btaf040_Supplementary_Data

## Data Availability

The code for running experiments is available at https://github.com/guydurant/toolboxsf. The processed data and Singularity containers for running the models are available at https://zenodo.org/records/8410136.
