# Supplementary material for: Robustly interrogating machine learning-based scoring functions: what are they learning?
Source: Bioinformatics. 2025 Jan 28;41(2):btaf040. doi: 10.1093/bioinformatics/btaf040 (PMC11821266; doi:10.1093/bioinformatics/btaf040)
Supplement: btaf040_Supplementary_Data [file btaf040_supplementary_data.zip › 5699c_toolboxsf_bioinformatics_supplementary_information_confirmation-2.pdf]

Supplementary Information: Robustly interrogating machine learning-based scoring functions: what are they learning?

Guy Durant, Fergus Boyles, Kristian Birchall, Brian Marsden and Charlotte M. Deane

1 Complex Type

1.1 Protein structure selection methodology

For the Redocked set, ligands were simply redocked using Smina back into their cognate structure. As CASF 2016 consists of 5 hand-picked different complex structures with different ligands bound for the same protein type, e.g. HIV protease, for 57 different protein types, there is variability within the conformation for each protein allowing cross-docking into alternative structures within the test set. To generate two classes of cross-docked structures, we aligned the pocket files, provided by PDBBind, using TM-Align (Zhang and Skolnick, 2005) in each set of 5 conformations and calculated TM-Score (Zhang and Skolnick, 2004) for each alignment. The highest TM-Score between a pocket and another pocket within the set was considered the best quality structure for cross-docking (Cross-docked (Best)), and the lowest TM-Score was chosen as the worst quality structure for cross-docking (Cross-docked (Worst)). The original ligand was docked at the site of the cognate ligand for the "best" and "worst" structures.

Apo structures for each cluster were identified by hand from the PDB. The PDB ID for the apo structure and their corresponding CASF 2016 PDB IDs are listed below. Only proteins that had no ligand bound in the active site and had 100% sequence identity for one of the five conformations in the set of 57 in CASF 2016 were considered. As the conformations in each set in CASF 2016 are not 100% sequence identical, we could not guarantee 100% sequence identical apo structure for each structure in CASF 2016. Of the 57 sets in CASF 2016, only 46 had a suitable apo structure so the final test set was only 230 complexes in size. For each complex, we aligned the apo structure to the original structure and then docked the ligand into the pocket of the apo structure.

For the Alphafold 2 version of CASF 2016, we predicted structures using AlphaFold2 (Jumper *et al.*, 2021) for monomers, and AlphafoldMultimer v2.1 (Evans *et al.*, 2022) for proteins consisting of multiple polypeptides. Predictions were run as described in the original publication (Jumper *et al.*, 2021), including sequences from UniRef (Suzek *et al.*, 2007) as well as BFD (Jumper *et al.*, 2021) and Mgnify (Mitchell *et al.*, 2019). To emulate a realistic blind prediction scenario, we did not include any templates in the prediction, although of course a notable fraction of the targets will have been part of AlphaFold 2's training set. The ligand was then docked into the aligned AlphaFold structure. All CASF 2016 structures could be successfully predicted, except one: PDB:1YDR. Finally, for the Wrong Protein set, the ligand was docked into a randomly chosen protein not from the same set but still from the CASF 2016 set. We also include visualisations in 3D and 2D of how these different complex types impact the docking pose and so the interactions formed for the PDB:1E66.

1.2 PDB IDs of CASF 2016 proteins and their respective Apo PDB ID

Holo	Apo
2xb8 3n76 3n7a 3n86 4ciw	2dhq
1nc1 1nc3 1y6r 4f2w 4f3c	1z5p
3u8k 3u8n 3zdg 4qac 3wtj	3sq9
1e66 1gpk 1gpn 1h22 1h23	7b38
2wvt 2xii 4j28 4jfs 4pcs	4j27
1ps3 3d4z 3dx1 3dx2 3ejr	3bub
1z95 3b5r 3b65 3b68 3g0w	None
3qqs 3r88 3twp 4gkm 4owm	3qr9
2fxs 2iwx 2vw5 2wer 2yge	1ah6
2cbv 2cet 2j78 2j7h 2wbg	1od0
2r9w 3gr2 3gv9 4jxs 4kz6	6t3d
3g2z 3g31 4de1 4de2 4de3	2p74
3nq9 3ueu 3uev 3uew 3uex	1b8e
2vkm 3rsx 3udh 4djv 4gid	2zhv
1k1i 1o3f 1uto 3gy4 4abg	5mnz
3p5o 3u5j 4lzs 4ogj 4wiv	4lyi
3ui7 3uuo 4llx 5c28 5c2h	2oup
1q8t 1q8u 1ydr 1ydt 3ag9	None
2weg 3dd0 3kwa 3ryj 4jsz	3ks3
3nw9 3oe4 3oe5 3ozs 3ozt	4pym
1pxn 2fvd 2xnb 3pxf 4eor	4ek3
4agn 4agp 4agq 5a7b 5aba	6shz
3arp 3arq 3aru 3arv 3ary	3b8s
1lpg 1mq6 1z6e 2xbv 2y5h	1hcg
4cr9 4cra 4crc 4ty7 4x6p	None
2zcq 2zcr 2zy1 3acw 4ea2	2zco
2v00 3prs 3pww 3uri 3wz8	5rdh
1qkt 2p15 2pog 2qe4 4mgd	None

Holo	Apo
1p1n 1p1q 1syi 2al5 4u4s	1fto
1vso 3fv1 3fv2 3gbb 4dld	None
3ebp 3g2n 3l7b 3syr 4eky	3e3l
1yc1 2xdl 2yki 3b27 3rlr	5j2v
3ao4 3zso 3zsx 3zt2 4cig	None
3ehy 3lka 3nx7 3tsk 4gr0	1os9
2zb1 3e92 3e93 4dli 4f9w	4e5b
2vvn 2w4x 2w66 2wca 2xj7	4ais
3coy 3coz 3ivg 4ddh 4ddk	3cov
2p4y 2yfe 3b1m 3fur 3u9q	6l8b
1a30 1eby 2qnq 3o9i 1g2k	3phv
1r5y 1s38 3gc5 3ge7 3rr4	4pun
1o0h 1u1b 1w4o 3d6q 3dxg	6etk
3cj4 3gnw 4eo8 4ih5 4ih7	1nb4
2wtv 3e5a 3myg 3uo4 3up2	6cpe
1nvq 2br1 2brb 3jvr 3jvs	1ia8
2c3i 3bgz 3jya 4k18 5dwr	1xqz
2wn9 2wnc 2x00 2xys 2ymd	2byn
3kr8 4j21 4j3l 4kzq 4kzu	3kr7
1qf1 1z9g 3fcq 4tmn 5tmn	2g4z
1bcu 1oyt 2zda 3bv9 3utu	4nzq
4bkt 4w9c 4w9h 4w9i 4w9l	3zrf
3f3a 3f3c 3f3d 3f3e 4mme	5jae
2v7a 3k5v 3mss 3pyy 4twp	None
3qgy 4m0y 4m0z 4qd6 4rfm	None
4e5w 4ivb 4ivc 4ivd 4k77	None
4e6q 4f09 4gfm 4hge 4jia	None
1bzc 2hb1 2qbp 2qbq 2qbr	5k9v
1c5z 1o5b 1owh 1sqa 3kgp	4dw2

1.3 Visualisation of different complex types for PDB:1E66 and their interactions

<table>
<tr><th>Complex Type</th><th>3D Structure</th><th>Interactions</th></tr>
<tr><td>Crystal</td><td>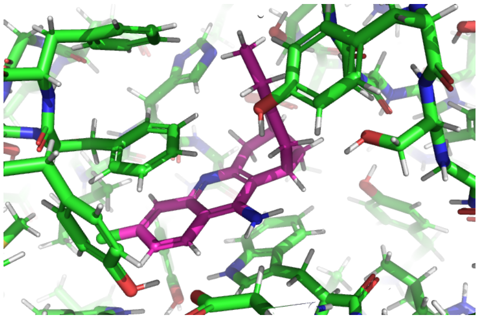</td><td>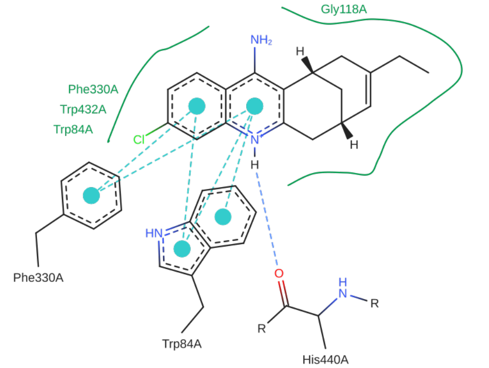
</td></tr>
<tr><td>Redocked</td><td>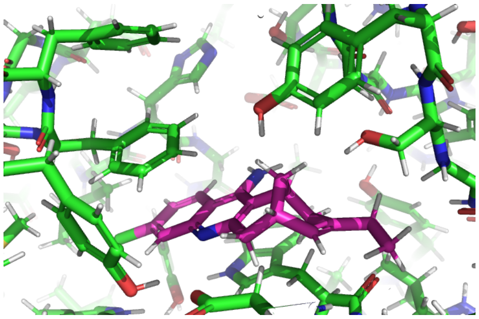</td><td>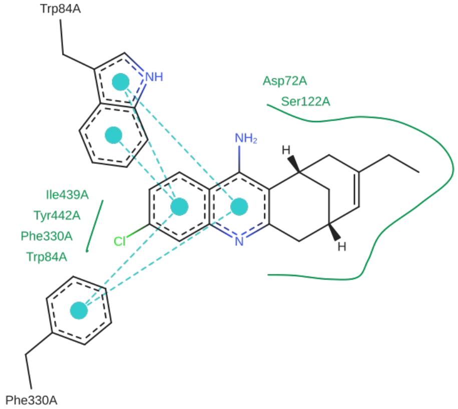
</td></tr>
<tr><td>Crossdocked (Best)</td><td>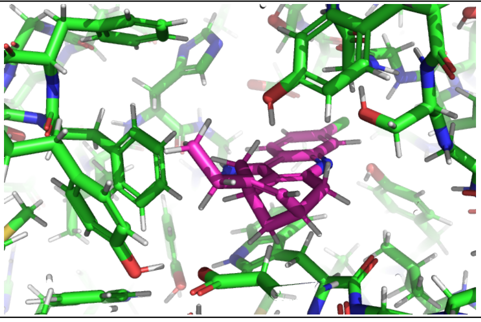</td><td>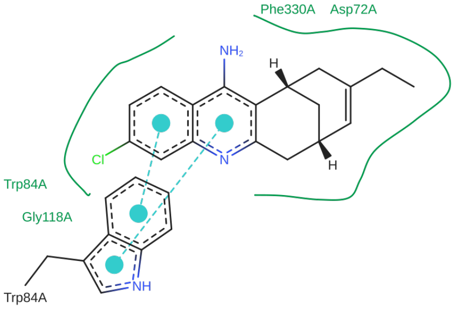
</td></tr>
<tr><td>Crossdocked (Worst)</td><td>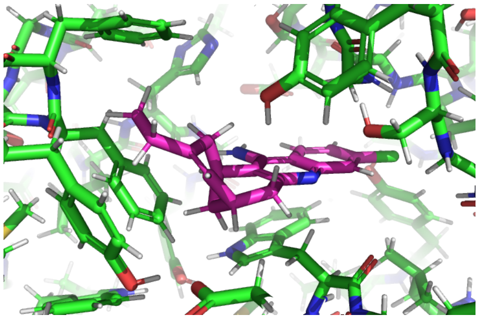</td><td>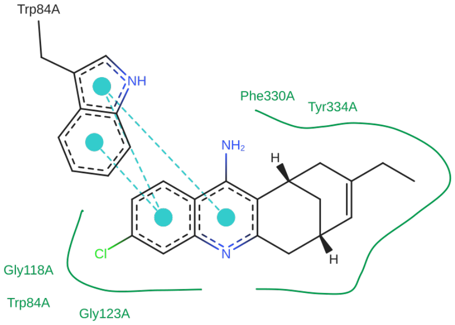
</td></tr>
</table>

Apo	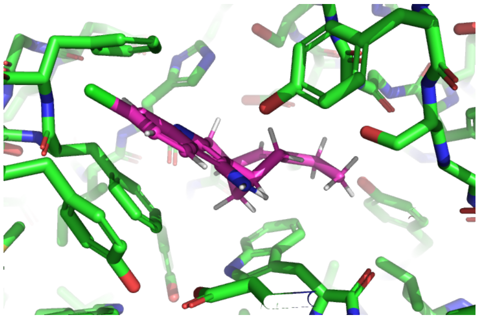	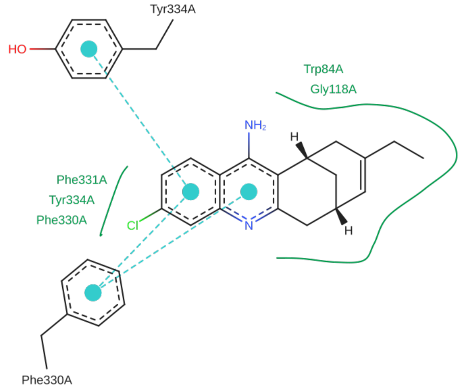
Alphafold 2	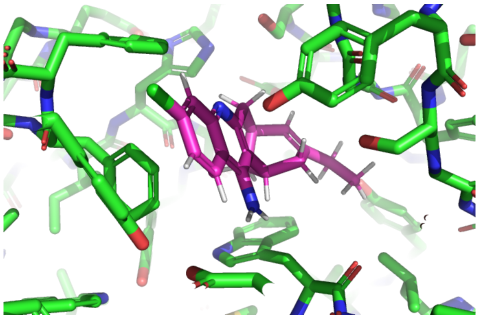	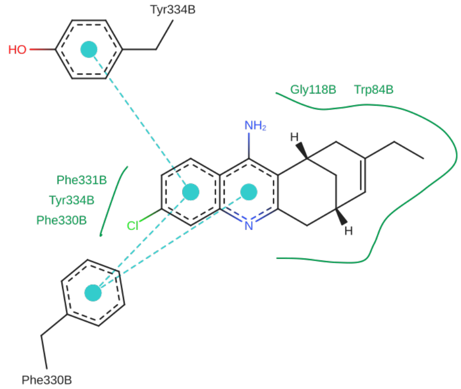
Wrong Protein	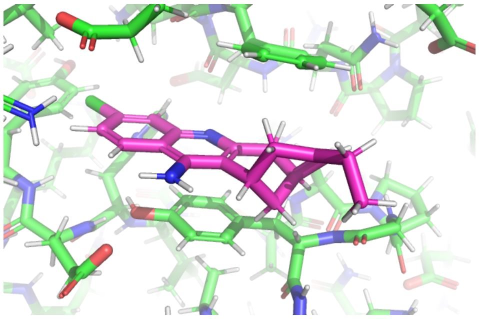	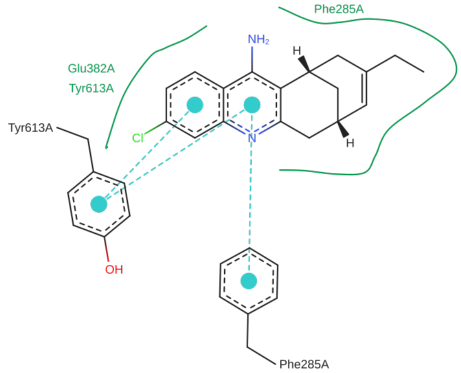

Table 3: 3D and 2D depictions of the protein-ligand complex PDB:1E66 for the different complex types of the Complex Type test sets. The 3D visualisations are at a fixed, consistent orientation and were made using PyMOL (Schrödinger, LLC, 2015). The 2D depiction of the ligand and the interactions with side chains were generated using PoseEdit (Diedrich *et al.*, 2023) These demonstrate the effect of docking into increasingly noisy protein structures on complex accuracy and the interactions.

2 Model implementation

2.1 Machine learning-based scoring function (MLBSF) modified implementations

MLBSF	Modification
RFScore (Ballester and Mitchell, 2010)	We reimplemented it with RandomForestRegressor, using the original hyperparameters, from the SciKit package (Pedregosa *et al.*, 2011) with features taken from the Open Drug Discovery Toolkit (ODDT) (Wójcikowski *et al.*, 2015) package. Although the hyperparameters could not be perfectly matched due to differences in the Random Forest implementations, it is unlikely this affected performance.
PointVS (Scantlebury *et al.*, 2023)	Required a validation set for early stopping; we used a random sample of 1000 data points from the training set.
Pafnucy (Stepniewska-Dziubinska *et al.*, 2018)	The original implementation used charges assigned by ChimeraX (Goddard *et al.*, 2018), however, due to its complexity as a dependency, we chose to set all charges to zero.
SIGN (Li *et al.*, 2021)	Like Pafnucy, also uses charges for the protein pocket from ChimeraX, so also had these features set to zero. It also required a validation set for early stopping; we used a random sample of 1000 data points from the training set.
OnionNet-2 (Wang *et al.*, 2021)	In the original implementation, they used a loss function that combined Pearson's R and root mean squared error (RMSE), PCC-RMSE. However, we found that this loss function often produced NaN losses during training so we used the RMSE loss function provided in their codebase instead. It also required a validation set for early stopping; we used a random sample of 1000 data points from the training set.

2.2 Hyperparameters of baseline models

LigandBias	ProteinBias	BothBias
Random Forest Regressor<br>• n_estimators = 362<br>• max_features = 0.51<br>• max_leaf_nodes = 2038	Random Forest Regressor<br>• n_estimators = 1447<br>• max_features = 0.32<br>• max_leaf_nodes = 5460	LGBMRegressor<br>• n_estimators = 205<br>• num_leaves = 291<br>• min_child_samples = 2<br>• learning_rate = 0.03<br>• log_max_bin = 9<br>• colsample_bytree = 0.6<br>• reg_alpha = 0.01<br>• reg_lambda = 0.01

2.3 Impact of protein pocket distance cutoff on accuracy for baseline models

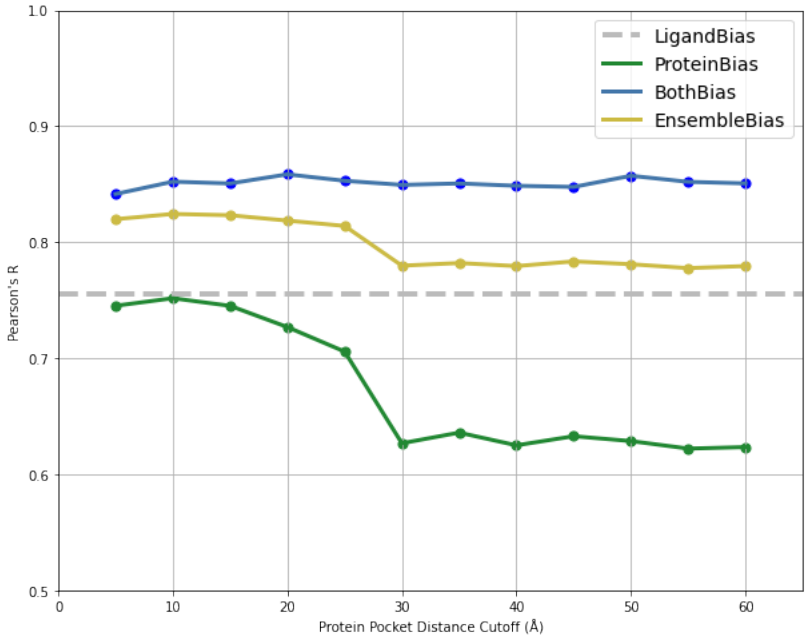

Figure 1: Pearson's R of baseline models on the CASF 2016 benchmark when changing the distance threshold, from any protein atom to any ligand atom, to include residues as being part of the protein pocket for featurisation.

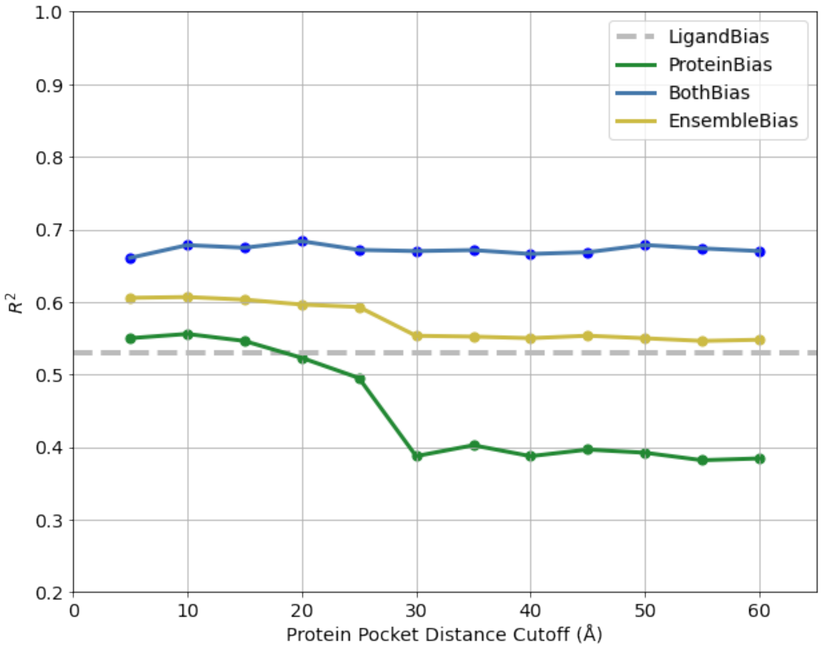

Figure 2: R^2 of baseline models on the CASF 2016 benchmark when changing the distance threshold, from any protein atom to any ligand atom, to include residues as being part of the protein pocket for featurisation.

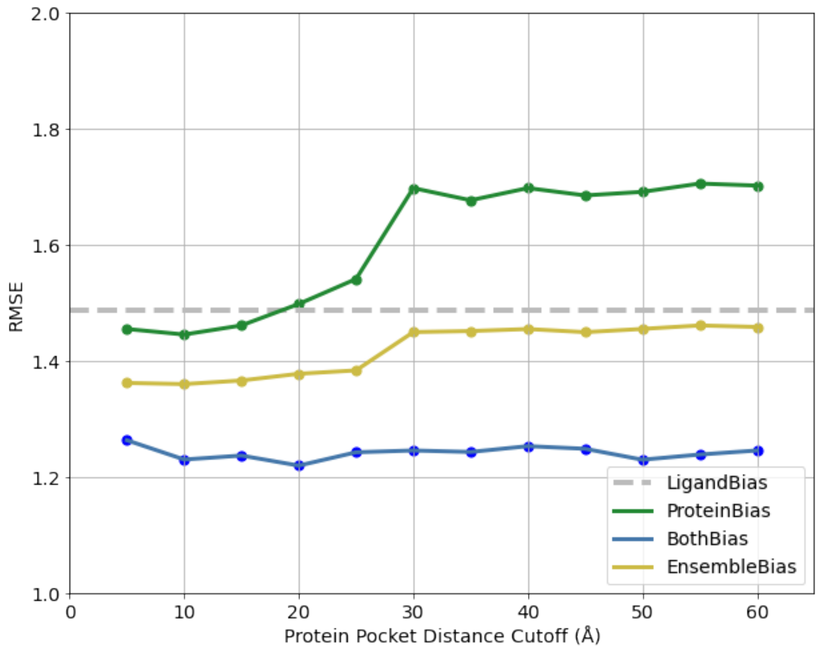

Figure 3: RMSE of baseline models on the CASF 2016 benchmark when changing the distance threshold, from any protein atom to any ligand atom, to include residues as being part of the protein pocket for featurisation.

3 Analysis of accuracy of scoring functions and baseline models on CASF 2016, 2019 Holdout, Peptides Holdout and 0 Ligand Bias

3.1 Further metrics for CASF 2016, 2019 Holdout, Peptides Holdout and 0 Ligand Bias

Method	CASF 2016		2019 Holdout		Peptides Holdout		0 Ligand Bias	
	R^2	RMSE	R^2	RMSE	R^2	RMSE	R^2	RMSE
LigandBias	$0.53_{\pm.07}$	$1.49_{\pm.12}$	$0.33_{\pm.04}$	$1.45_{\pm.05}$	$-0.37_{\pm.08}$	$1.81_{\pm.04}$	$-0.45_{\pm.24}$	$1.93_{\pm.14}$
ProteinBias	$0.55_{\pm.09}$	$1.46_{\pm.14}$	$0.34_{\pm.04}$	$1.44_{\pm.05}$	**$0.06_{\pm.04}$**	**$1.50_{\pm.05}$**	**$0.11_{\pm.11}$**	**$1.51_{\pm.14}$**
EnsembleBias	$0.60_{\pm.06}$	$1.37_{\pm.11}$	$0.44_{\pm.03}$	$1.33_{\pm.04}$	$0.01_{\pm.04}$	$1.54_{\pm.04}$	$-0.03_{\pm.13}$	$1.62_{\pm.12}$
BothBias	**$0.67_{\pm.05}$**	**$1.24_{\pm.10}$**	$0.45_{\pm.04}$	$1.32_{\pm.05}$	$-0.14_{\pm.06}$	$1.65_{\pm.04}$	$-0.10_{\pm.16}$	$1.68_{\pm.13}$
Smina	$0.27_{\pm.13}$	$1.86_{\pm.16}$	$-0.95_{\pm.23}$	$2.49_{\pm.11}$	$-2.31_{\pm.30}$	$2.82_{\pm.10}$	$-2.31_{\pm.81}$	$2.91_{\pm.24}$
RFScore	$0.60_{\pm.05}$	$1.37_{\pm.11}$	$0.41_{\pm.04}$	$1.36_{\pm.05}$	$-0.26_{\pm.07}$	$1.73_{\pm.04}$	$-0.13_{\pm.15}$	$1.70_{\pm.13}$
PointVS	$0.58_{\pm.06}$	$1.40_{\pm.10}$	$0.44_{\pm.04}$	$1.33_{\pm.04}$	$-0.03_{\pm.06}$	$1.57_{\pm.04}$	$-0.06_{\pm.15}$	$1.65_{\pm.13}$
Pafnucy	$0.51_{\pm.06}$	$1.51_{\pm.11}$	$0.35_{\pm.05}$	$1.44_{\pm.06}$	$-0.30_{\pm.08}$	$1.77_{\pm.04}$	$-0.16_{\pm.15}$	$1.73_{\pm.12}$
SIGN	**$0.67_{\pm.07}$**	**$1.24_{\pm.11}$**	$0.41_{\pm.05}$	$1.36_{\pm.05}$	$-0.03_{\pm.07}$	$1.57_{\pm.05}$	$-0.17_{\pm.18}$	$1.73_{\pm.14}$
OnionNet-2	$0.66_{\pm.06}$	$1.26_{\pm.10}$	**$0.49_{\pm.04}$**	**$1.28_{\pm.05}$**	$0.05_{\pm.05}$	$1.51_{\pm.04}$	$-0.04_{\pm.16}$	$1.64_{\pm.15}$

Table 4: R^2 and RMSE (in pK units) between predicted and true pK values for protein-ligand complexes for our baseline models (LigandBias, ProteinBias, EnsembleBias and BothBias), a non-ML-based scoring function (Smina) and five commonly used MLBSFs (RFScore, PointVS, Pafnucy, SIGN and OnionNet-2) on four benchmark datasets (CASF 2016, 2019 Holdout, Peptides Holdout and 0 Ligand Bias). See methods for further details of scoring functions and dataset creation. Error ranges represent the 95% confidence intervals from bootstrapped R^2 and RMSE (N=10000). The highest values are in bold and underlined, with any value within the highest values' confidence intervals underlined.

3.2 Peptides Holdout Analysis

To examine whether low performance on Peptides Holdout is due to their low drug-likeness (Bickerton *et al.*, 2012), we restricted the set to any peptide with 10 amino acids or less as 80% of oral peptide drugs were found to be within this range (Santos *et al.*, 2016). We show the distribution of the peptide lengths of the Peptide Holdout ligands in Figure 4 and show that this roughly halves the available peptides. Of the 2573 complexes, 1228 are below this threshold.

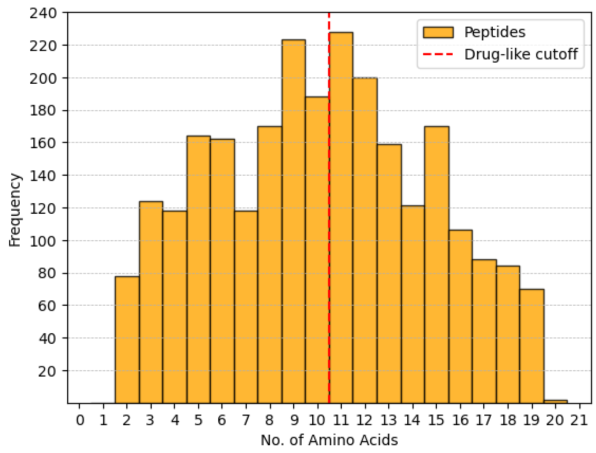

Figure 4: Histogram of the peptide amino acid lengths for the Peptide Holdout test set. The red line indicates the 10 amino acid threshold chosen from (Santos *et al.*, 2016)

Performance of all scoring functions did incrementally increase with this subset as shown in Table 5,

demonstrating that the longer peptides were harder to score. However, performance on the whole is still very low showing that they still have not learnt the biophysics necessary to score these peptide-protein complexes.

Method	Peptides Holdout <10 amino acids		
	r	R^2	RMSE
LigandBias	$0.31_{\pm.05}$	$-0.19_{\pm.10}$	$1.83_{\pm.06}$
ProteinBias	$0.42_{\pm.05}$	$0.15_{\pm.06}$	$\underline{1.54_{\pm.07}}$
EnsembleBias	$\underline{0.46_{\pm.05}}$	$\underline{0.11_{\pm.06}}$	$1.58_{\pm.06}$
BothBias	$0.43_{\pm.05}$	$0.02_{\pm.08}$	$1.66_{\pm.06}$
Smina	$0.20_{\pm.05}$	$-1.60_{\pm.33}$	$2.70_{\pm.15}$
RFScore	$0.40_{\pm.05}$	$-0.02_{\pm.08}$	$1.69_{\pm.06}$
PointVS	$0.44_{\pm.05}$	$0.09_{\pm.08}$	$1.59_{\pm.06}$
Pafnucy	$0.43_{\pm.05}$	$-0.11_{\pm.09}$	$1.76_{\pm.06}$
SIGN	$\underline{\mathbf{0.49_{\pm.04}}}$	$\underline{\mathbf{0.18_{\pm.07}}}$	$\underline{\mathbf{1.51_{\pm.06}}}$
OnionNet-2	$\underline{0.46_{\pm.05}}$	$\underline{0.13_{\pm.07}}$	$\underline{1.56_{\pm.06}}$

Table 5: Pearson's R (r), R^2 and RMSE (in pK units) between predicted and true pK values for protein-ligand complexes for our baseline models (LigandBias, ProteinBias, EnsembleBias and BothBias), a non-ML-based scoring function (Smina) and five commonly used MLBSFs (RFScore, PointVS, Pafnucy, SIGN and OnionNet-2) on Peptides Holdout crystal structures when restricted to peptides of length 10 amino acids or less. Error ranges represent the 95% confidence intervals from bootstrapped Pearson's R, R^2 and RMSE (N=10000). The highest values are in bold and underlined, with any value within the highest values' confidence intervals underlined.

4 Accuracy of scoring functions and baseline models on protein family hold-out clusters

All the benchmarks investigated in the paper do not reflect a realistic drug discovery scenario as they measure accuracy across many different protein families at once instead of the typical process of screening against a single protein target. We also created the Protein Family Out Benchmarks test sets to measure scoring function accuracy on specific protein families. We clustered the PDBBind dataset, using 90% sequence identity clusters from the PDB (RCBS, 2023) and took any cluster that had more than 100 data points as separate test sets to simulate the screening of a single protein target. The 100 data point size limit ensured there were sufficient data points to evaluate scoring function accuracy. We then trained a new version of each scoring function without each cluster and tested them on the held-out cluster to simulate the scenario of these scoring functions being used on a novel protein target in a virtual screen.

These hold-out tests demonstrate the overall trend, as shown in Figures 5, 6 and 7, that the scoring functions do not vary greatly in the accuracy they achieve, with larger differences in the average ability between protein families than between model accuracy on the same family. The baseline models also follow this trend suggesting that the reason for these differences is probably due to the protein families having different similarities to the training dataset rather than any deeper insight into the scoring functions' performance.

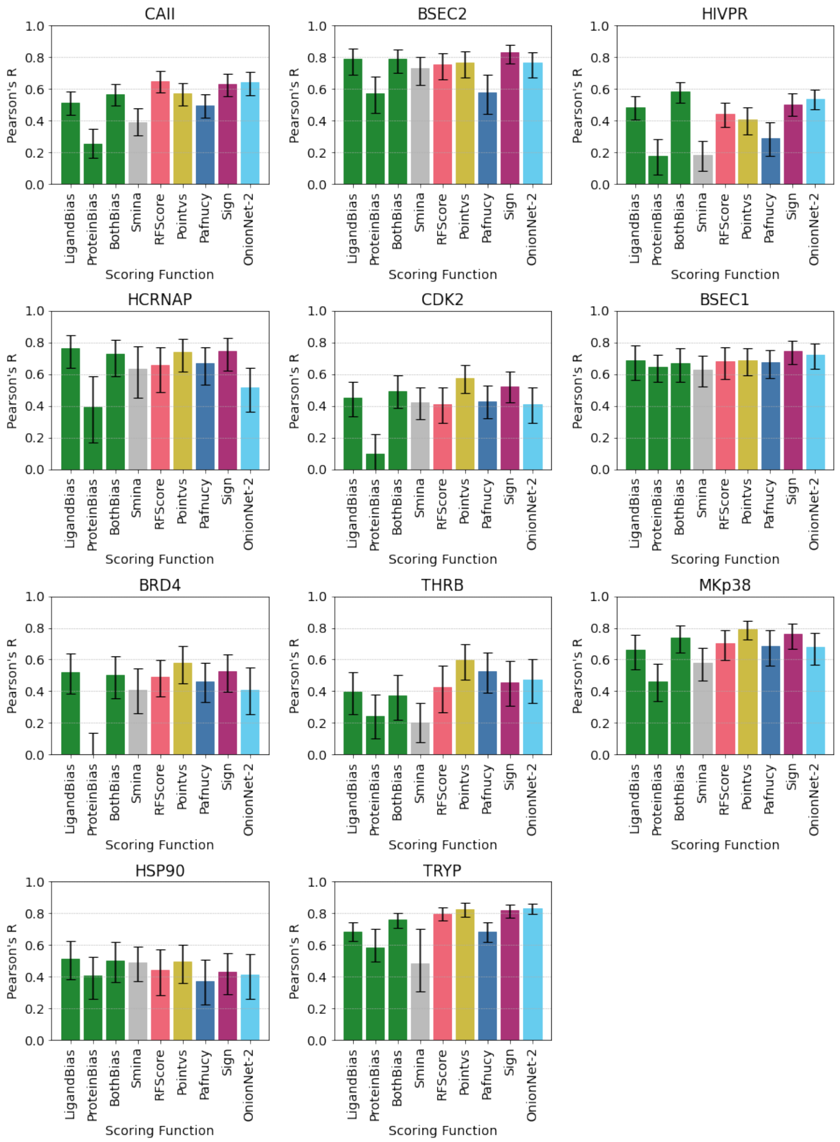

Figure 5: Pearson's R between predicted and true pK values for protein-ligand complexes for our baseline models (Ligand Bias, Protein Bias and Both Bias), a non-ML-based scoring function (Smina) and five commonly used MLBSFs (RFScore, PointVS, Pafnucy, SIGN and OnionNet-2) for eleven protein family hold-out clusters. These eleven families are Carbonic Anhydrase II (CAII), Beta-secretase (BSEC2), HIV protease (HIVPR), Hepatitis C Virus RNA-polymerase (HCRNAP), Cyclin-dependent kinase 2 (CKD2), Beta-secretase (BSEC1), Bromodomain-containing protein 4 (BRD4), Thrombin (THRB), MAP Kinase p28 (MKp38), Heat Shock Protein 90 (HSP90) and Trypsin (TRYP). Error bars represent the 95% confidence intervals from bootstrapped Pearson's R (N=10000).

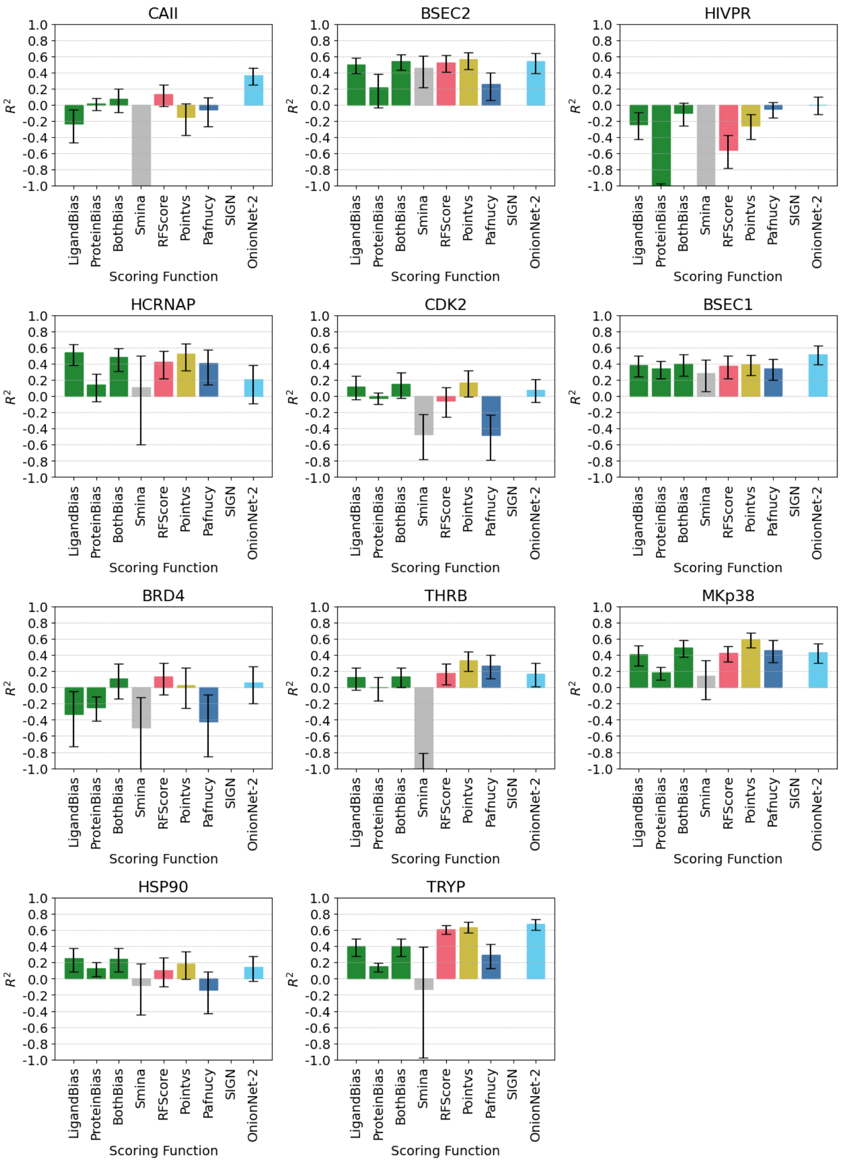

Figure 6: R^2 between predicted and true pK values for protein-ligand complexes for our baseline models (Ligand Bias, Protein Bias and Both Bias), a non-ML-based scoring function (Smina) and five commonly used MLBSFs (RFScore, PointVS, Pafnucy, SIGN and OnionNet-2) for eleven protein family hold-out clusters. These eleven families are Carbonic Anhydrase II (CAII), Beta-secretase (BSEC2), HIV protease (HIVPR), Hepatitis C Virus RNA-polymerase (HCRNAP), Cyclin-dependent kinase 2 (CKD2), Beta-secretase (BSEC1), Bromodomain-containing protein 4 (BRD4), Thrombin (THRB), MAP Kinase p28 (MKp38), Heat Shock Protein 90 (HSP90) and Trypsin (TRYP). Error bars represent the 95% confidence intervals from bootstrapped R^2 (N=10000).

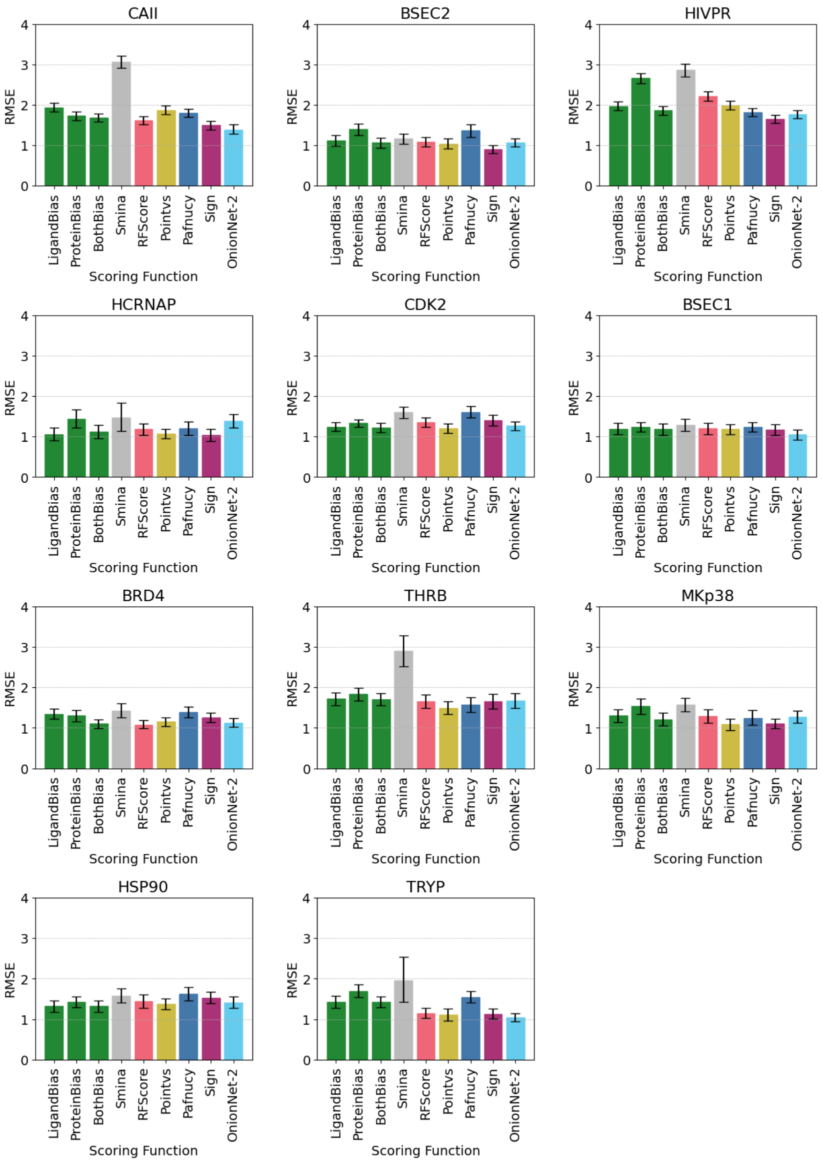

Figure 7: RMSE between predicted and true pK values for protein-ligand complexes for our baseline models (Ligand Bias, Protein Bias and Both Bias), a non-ML-based scoring function (Smina) and five commonly used MLBSFs (RFScore, PointVS, Pafnucy, SIGN and OnionNet-2) for eleven protein family hold-out clusters. These eleven families are Carbonic Anhydrase II (CAII), Beta-secretase (BSEC2), HIV protease (HIVPR), Hepatitis C Virus RNA-polymerase (HCRNAP), Cyclin-dependent kinase 2 (CKD2), Beta-secretase (BSEC1), Bromodomain-containing protein 4 (BRD4), Thrombin (THRB), MAP Kinase p28 (MKp38), Heat Shock Protein 90 (HSP90) and Trypsin (TRYP). Error bars represent the 95% confidence intervals from bootstrapped RMSE (N=10000).

5 Further metrics for scoring functions and baselines models on different complex types of CASF 2016

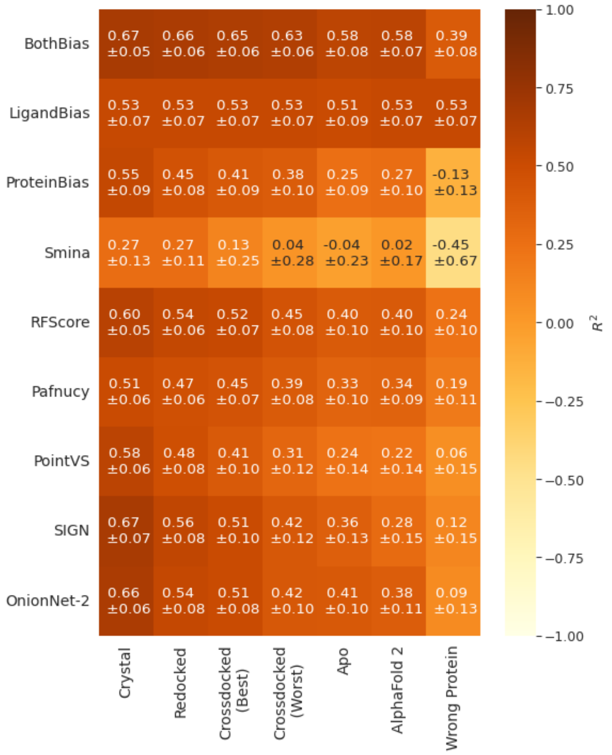

Figure 8: R^2 between predicted and true pK values for protein-ligand complexes for our baseline models (LigandBias, ProteinBias and BothBias), a non-ML-based scoring function (Smina) and five commonly used MLBSFs (RFScore, PointVS, Pafnucy, SIGN and OnionNet-2) on alternate CASF 2016 complex type test sets. Errors are the 95% confidence intervals from the bootstrapped R^2

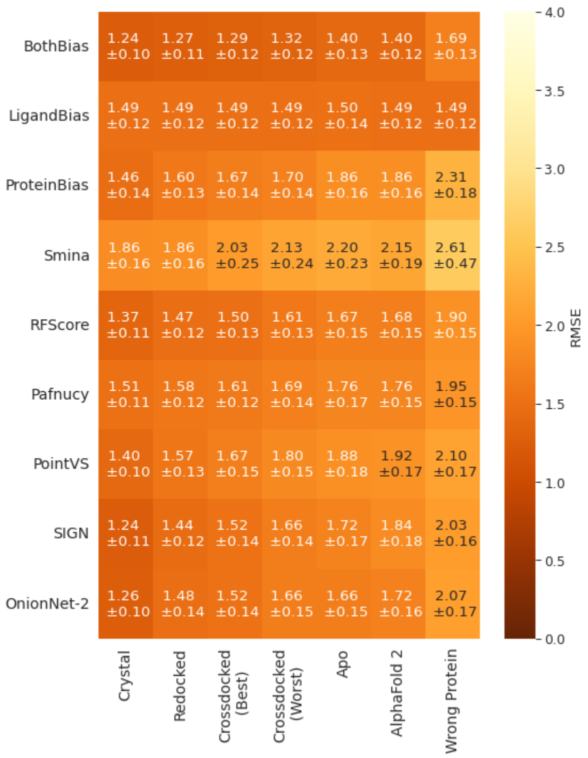

Figure 9: RMSE between predicted and true pK values for protein-ligand complexes for our baseline models (LigandBias, ProteinBias and BothBias), Pafnucy and Smina on different accuracy poses of CASF 2016 complexes. Accuracy on the crystal structures of CASF 2016 is shown as a dashed black line. Errors are the 95% confidence intervals from the bootstrapped RMSE (N=10000).

6 Further scoring functions and baseline models on differing docking accuracy versions of CASF 2016

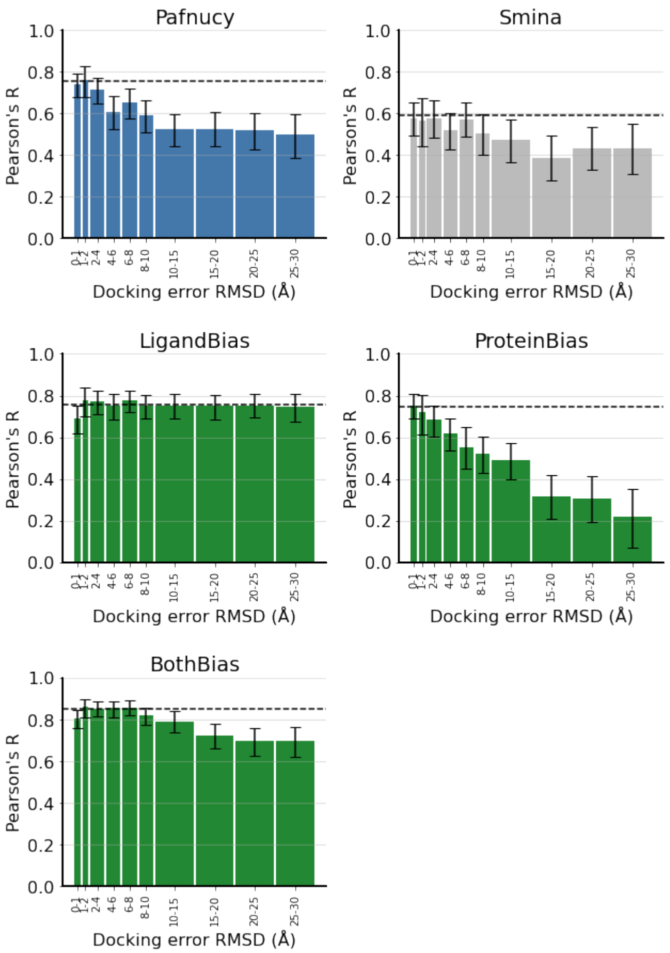

Figure 10: Pearson's R between predicted and true pK values for protein-ligand complexes for our baseline models (LigandBias, ProteinBias and BothBias), Pafnucy and Smina on different accuracy poses of CASF 2016 complexes. Accuracy on the crystal structures of CASF 2016 is shown as a dashed black line. Errors are the 95% confidence intervals from the bootstrapped Pearson's R (N=10000).

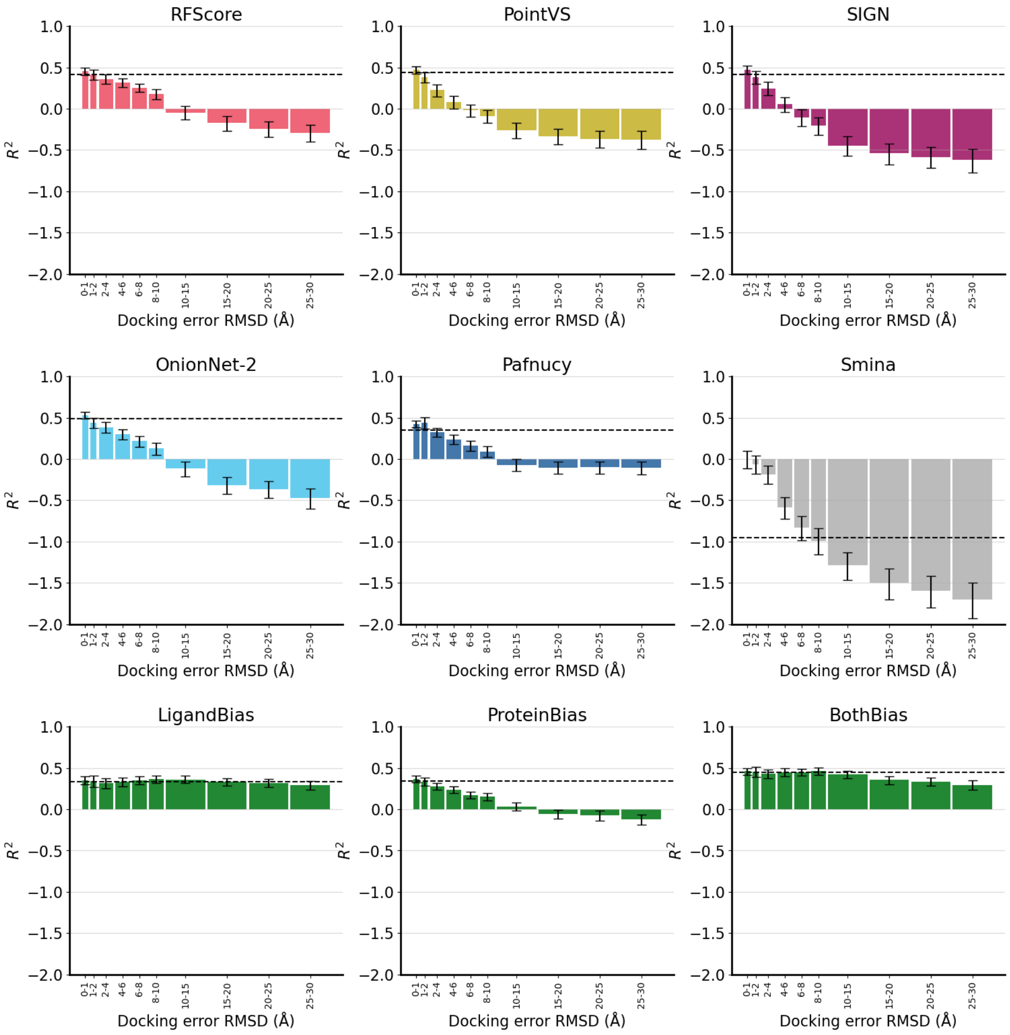

Figure 11: R^2 between predicted and true pK values for protein-ligand complexes for our baseline models (LigandBias, ProteinBias and BothBias), a non-ML-based scoring function (Smina) and five commonly used MLBSFs (RFScore, PointVS, Pafnucy, SIGN and OnionNet-2), on different accuracy poses of CASF 2016 complexes. Accuracy on the crystal structures of CASF 2016 is shown as a dashed black line. Errors are the 95% confidence intervals from the bootstrapped R^2 (N=10000).

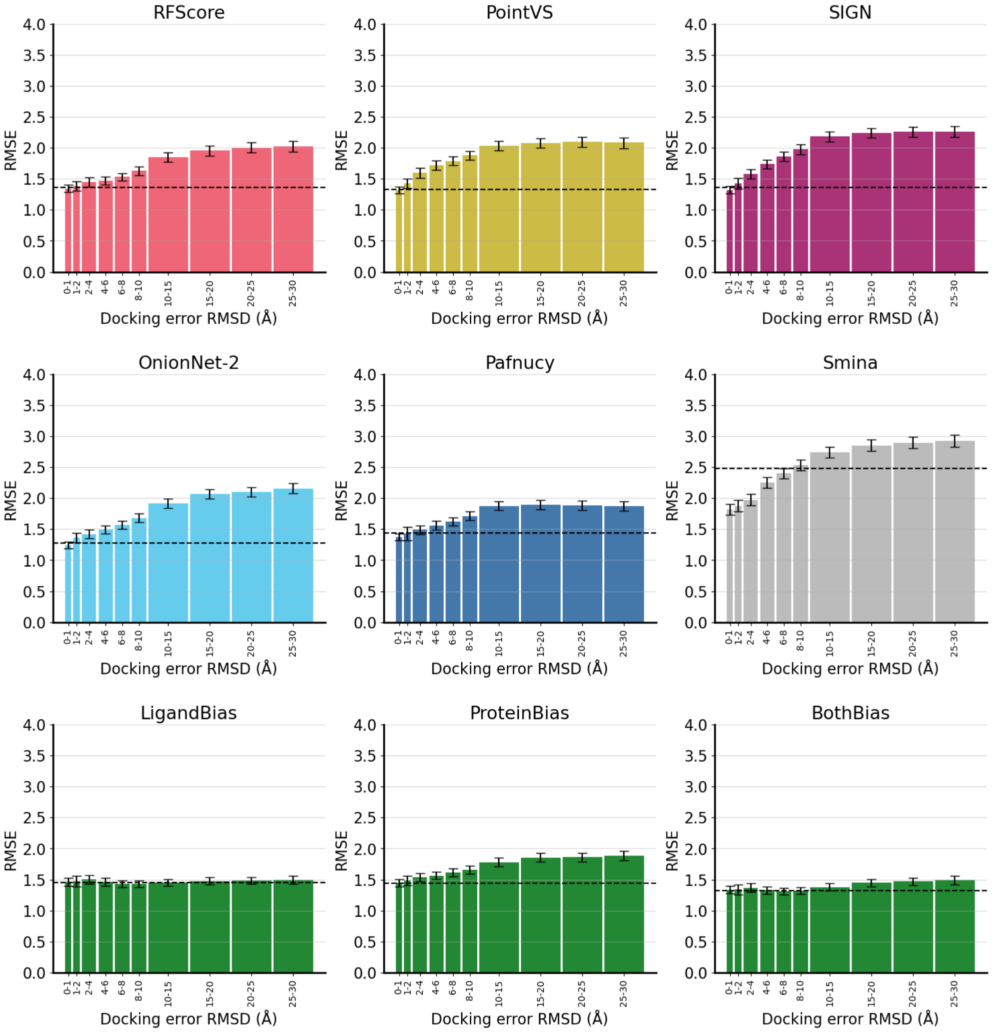

Figure 12: RMSE between predicted and true pK values for protein-ligand complexes for our baseline models (LigandBias, ProteinBias and BothBias), a non-ML-based scoring function (Smina) and five commonly used MLBSFs (RFScore, PointVS, Pafnucy, SIGN and OnionNet-2), on different accuracy poses of CASF 2016 complexes. Accuracy on the crystal structures of CASF 2016 is shown as a dashed black line. Errors are the 95% confidence intervals from the bootstrapped RMSE (N=10000).

7 Accuracy of scoring functions and baseline models on differing docking accuracy versions of 2019 Holdout

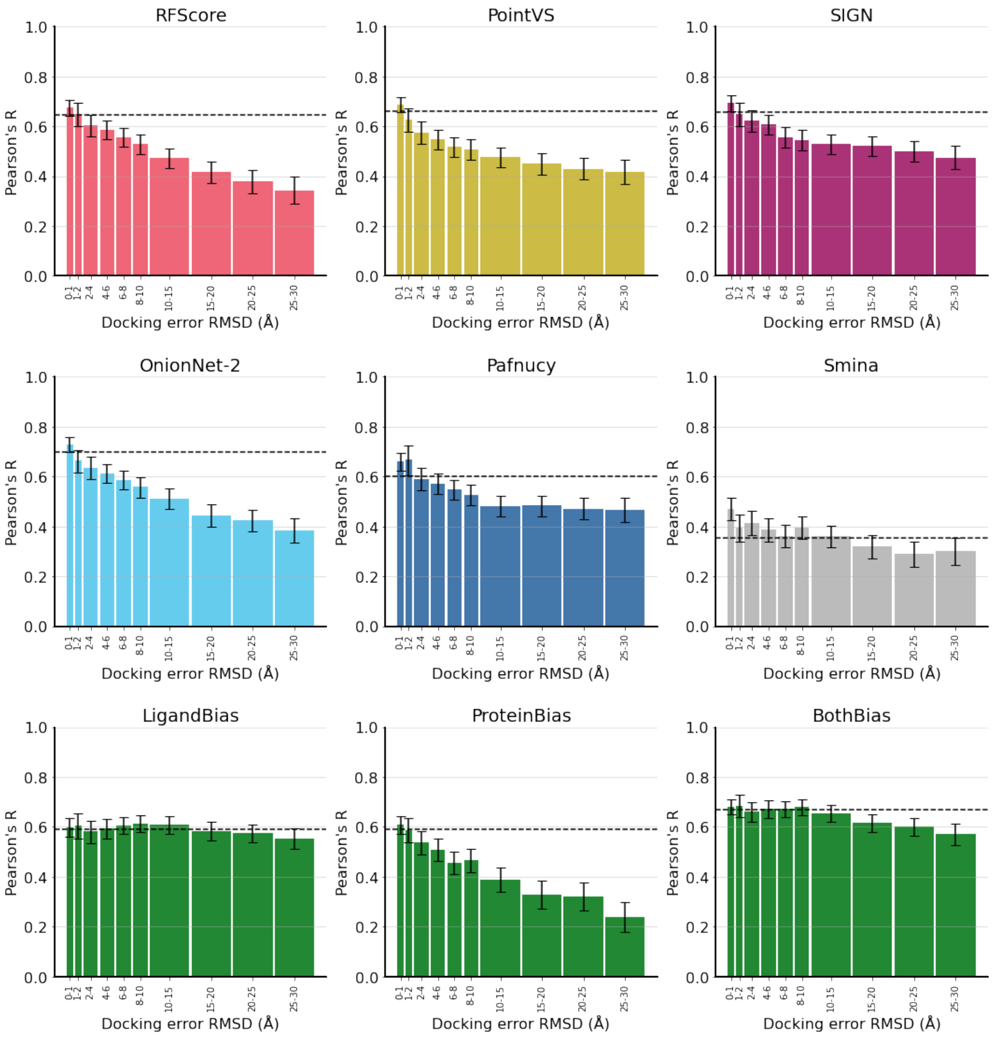

Figure 13: Pearson's R between predicted and true pK values for protein-ligand complexes for our baseline models (LigandBias, ProteinBias and BothBias), a non-ML-based scoring function (Smina) and five commonly used MLBSFs (RFScore, PointVS, Pafnucy, SIGN and OnionNet-2), on different accuracy poses of 2019 Holdout complexes. Accuracy on the crystal structures of 2019 Holdout is shown as a dashed black line. Errors are the 95% confidence intervals from the bootstrapped Pearson's R (N=10000).

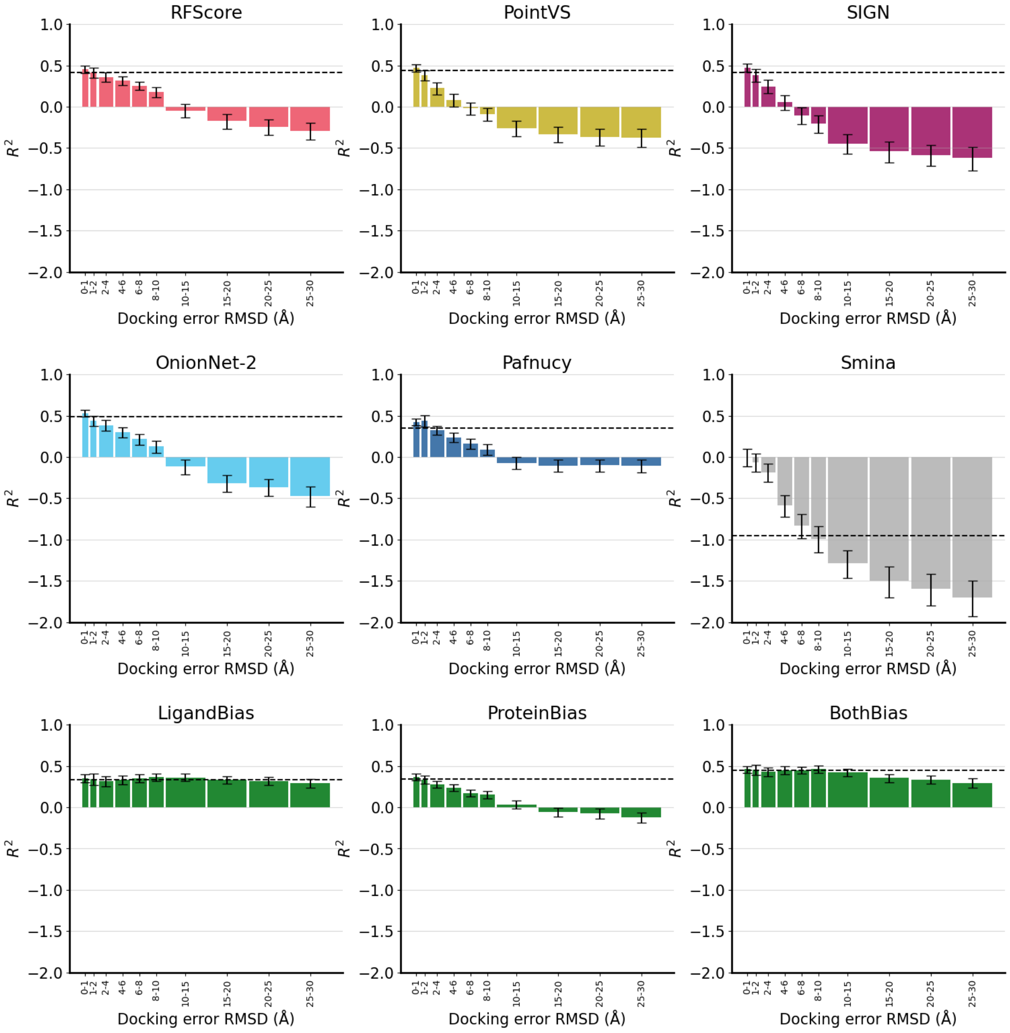

Figure 14: R^2 between predicted and true pK values for protein-ligand complexes for our baseline models (LigandBias, ProteinBias and BothBias), a non-ML-based scoring function (Smina) and five commonly used MLBSFs (RFScore, PointVS, Pafnucy, SIGN and OnionNet-2), on different accuracy poses of 2019 Holdout complexes. Accuracy on the crystal structures of 2019 Holdout is shown as a dashed black line. Errors are the 95% confidence intervals from the bootstrapped R^2 (N=10000).

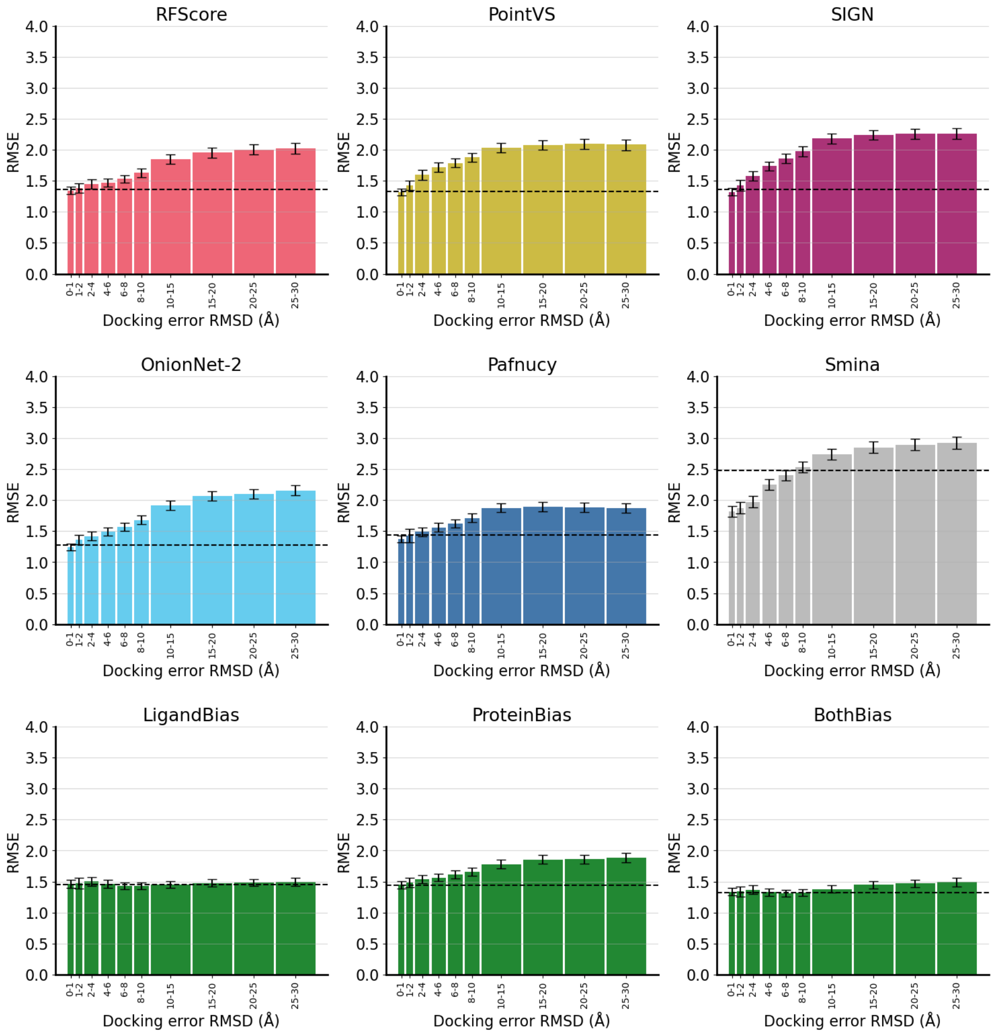

Figure 15: RMSE between predicted and true pK values for protein-ligand complexes for our baseline models (LigandBias, ProteinBias and BothBias), a non-ML-based scoring function (Smina) and five commonly used MLBSFs (RFScore, PointVS, Pafnucy, SIGN and OnionNet-2), on different accuracy poses of 2019 Holdout complexes. Accuracy on the crystal structures of 2019 Holdout is shown as a dashed black line. Errors are the 95% confidence intervals from the bootstrapped RMSE (N=10000).

8 Accuracy of scoring functions and baseline models on differing docking accuracy versions of 0 Ligand Bias

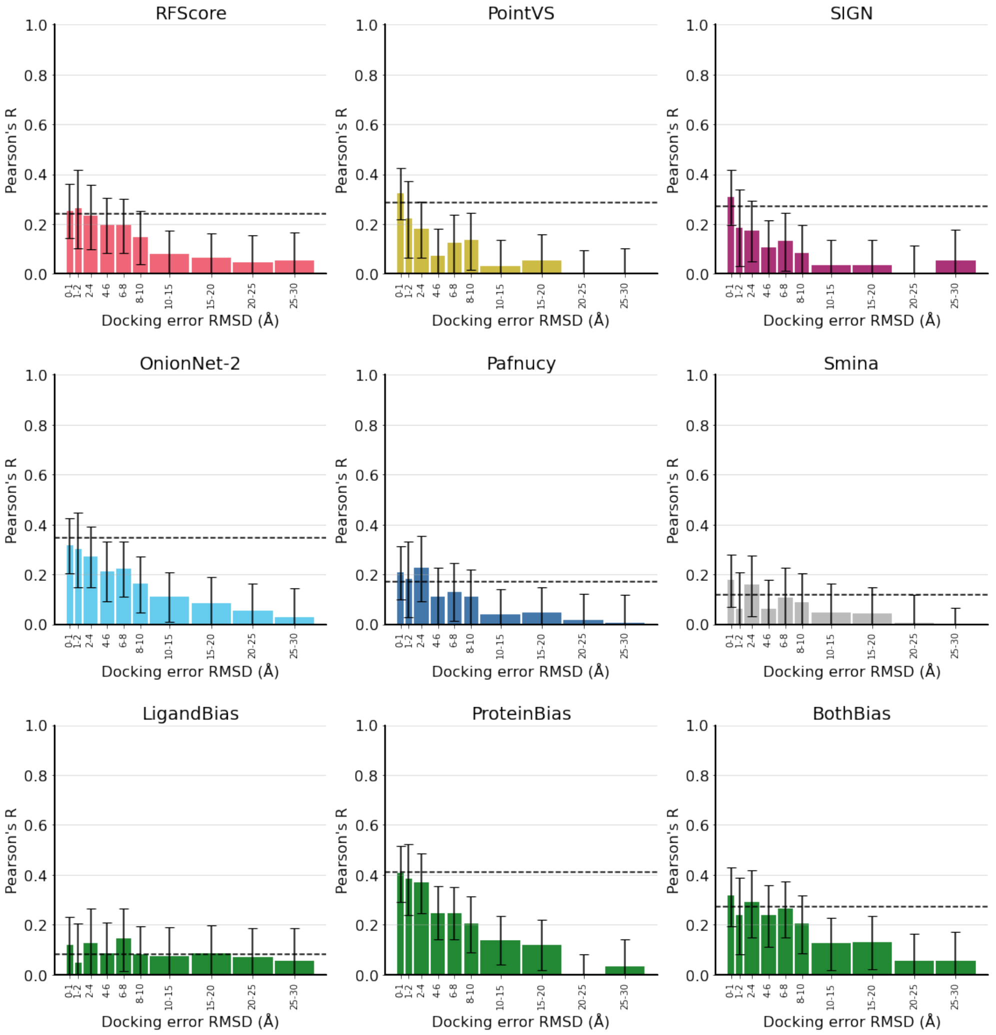

Figure 16: Pearson's R between predicted and true pK values for protein-ligand complexes for our baseline models (LigandBias, ProteinBias and BothBias), a non-ML-based scoring function (Smina) and five commonly used MLBSFs (RFScore, PointVS, Pafnucy, SIGN and OnionNet-2), on different accuracy poses of 0 Ligand Bias complexes. Accuracy on the crystal structures of 0 Ligand Bias is shown as a dashed black line. Errors are the 95% confidence intervals from the bootstrapped Pearson's R (N=10000).

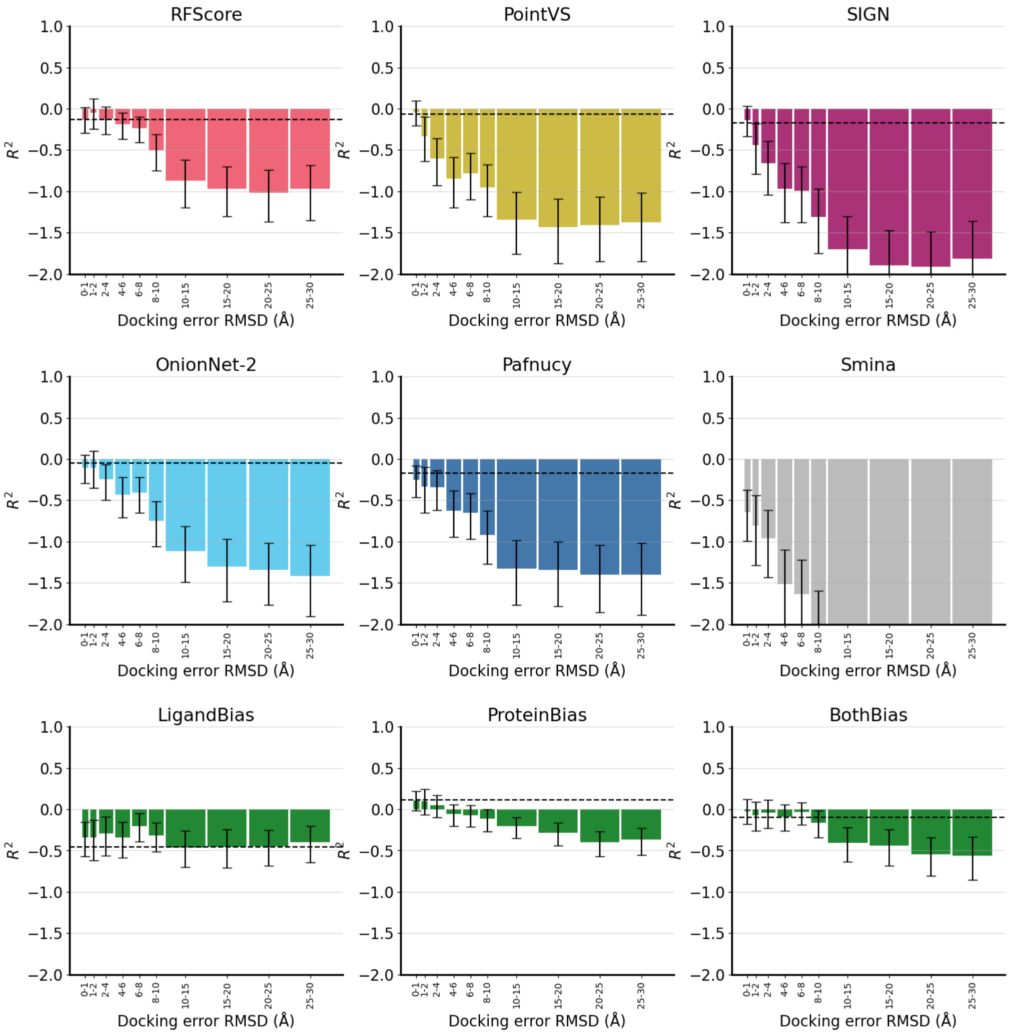

Figure 17: R^2 between predicted and true pK values for protein-ligand complexes for our baseline models (LigandBias, ProteinBias and BothBias), a non-ML-based scoring function (Smina) and five commonly used MLBSFs (RFScore, PointVS, Pafnucy, SIGN and OnionNet-2), on different accuracy poses of 0 Ligand Bias complexes. Accuracy on the crystal structures of 0 Ligand Bias is shown as a dashed black line. Errors are the 95% confidence intervals from the bootstrapped R^2 (N=10000).

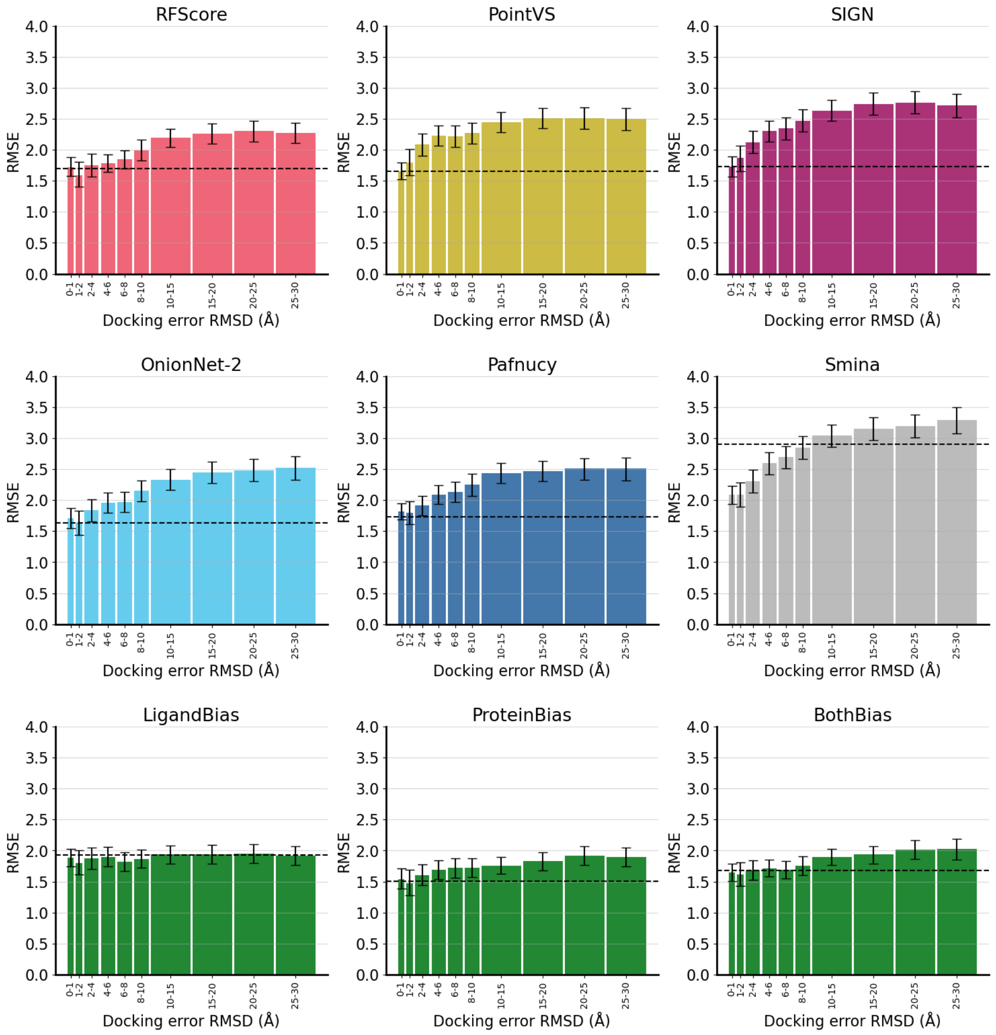

Figure 18: RMSE between predicted and true pK values for protein-ligand complexes for our baseline models (LigandBias, ProteinBias and BothBias), a non-ML-based scoring function (Smina) and five commonly used MLBSFs (RFScore, PointVS, Pafnucy, SIGN and OnionNet-2), on different accuracy poses of 0 Ligand Bias complexes. Accuracy on the crystal structures of 0 Ligand Bias is shown as a dashed black line. Errors are the 95% confidence intervals from the bootstrapped RMSE (N=10000).

9 Accuracy of scoring functions and baseline models on progressively displaced ligands of CASF 2016

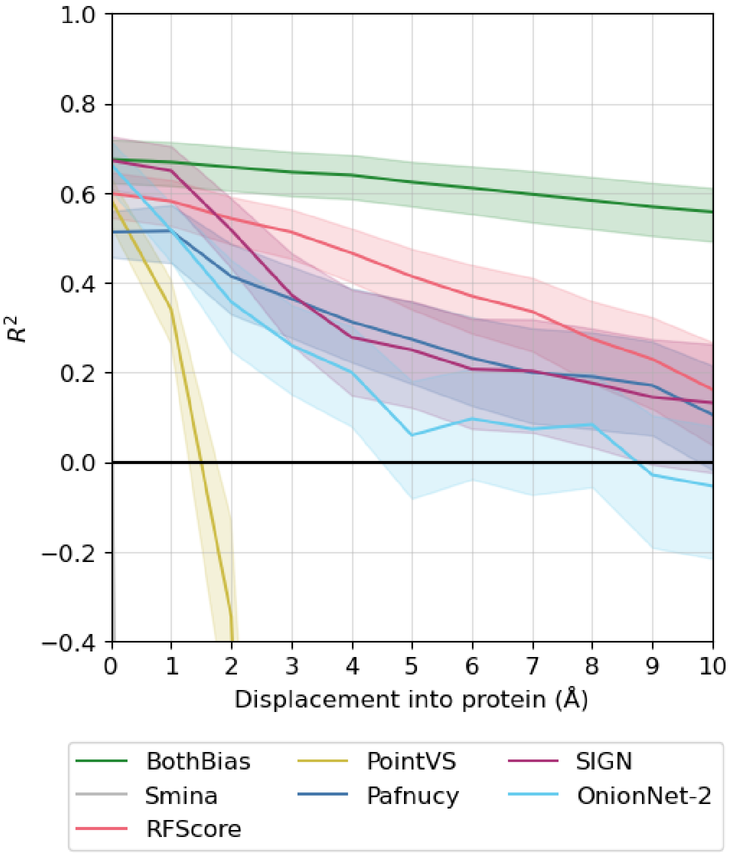

Figure 19: R^2 between predicted and true pK values for protein-ligand com-plexes for our baseline model (BothBias), a non-ML-based scoring function (Smina) and five commonly used MLBSFs (RFScore, PointVS, Pafnucy, SIGN and OnionNet-2), on progressively displaced ligands into the protein originally from CASF 2016 crystal structures. Errors are the 95% confidence intervals from the bootstrapped R^2 (N=10000).

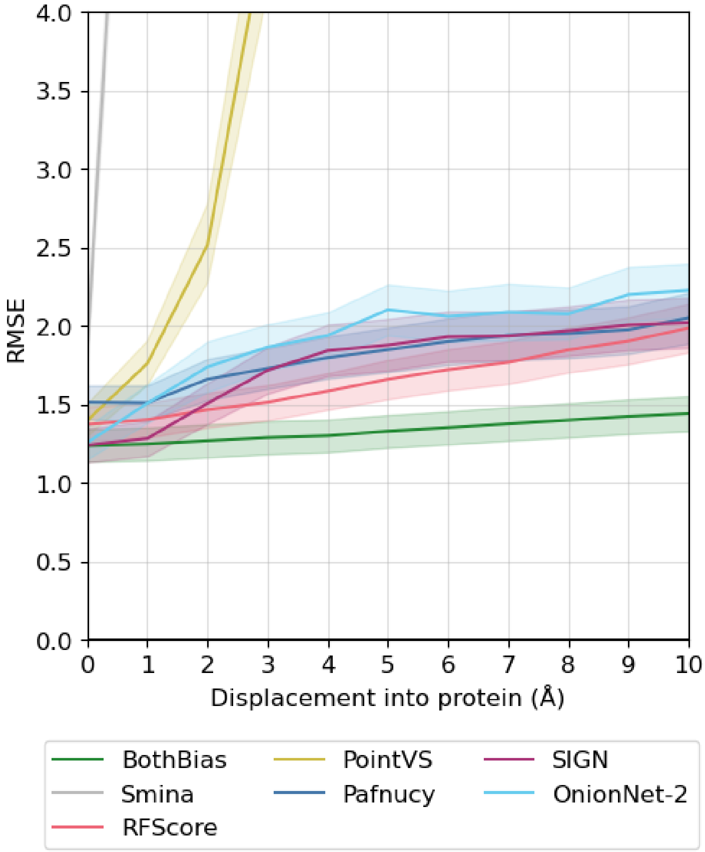

Figure 20: RMSE between predicted and true pK values for protein-ligand com-plexes for our baseline model (BothBias), a non-ML-based scoring function (Smina) and five commonly used MLBSFs (RFScore, PointVS, Pafnucy, SIGN and OnionNet-2), on progressively displaced ligands into the protein originally from CASF 2016 crystal structures. Errors are the 95% confidence intervals from the bootstrapped RMSE (N=10000).

10 Accuracy of scoring functions and baseline models on progressively displaced ligands of 2019 Holdout

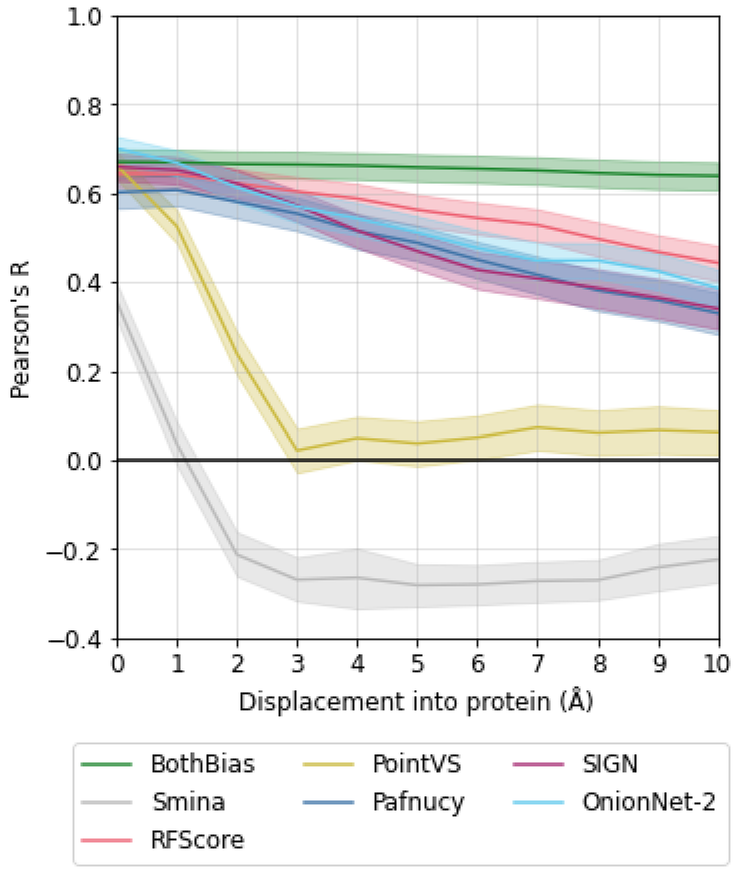

Figure 21: Pearson's R between predicted and true pK values for protein-ligand com- plexes for our baseline model (BothBias), a non-ML-based scoring function (Smina) and five commonly used MLBSFs (RFScore, PointVS, Pafnucy, SIGN and OnionNet-2), on progressively displaced ligands into the protein originally from 2019 Holdout crystal structures. Errors are the 95% confidence intervals from the bootstrapped Pearson's R (N=10000).

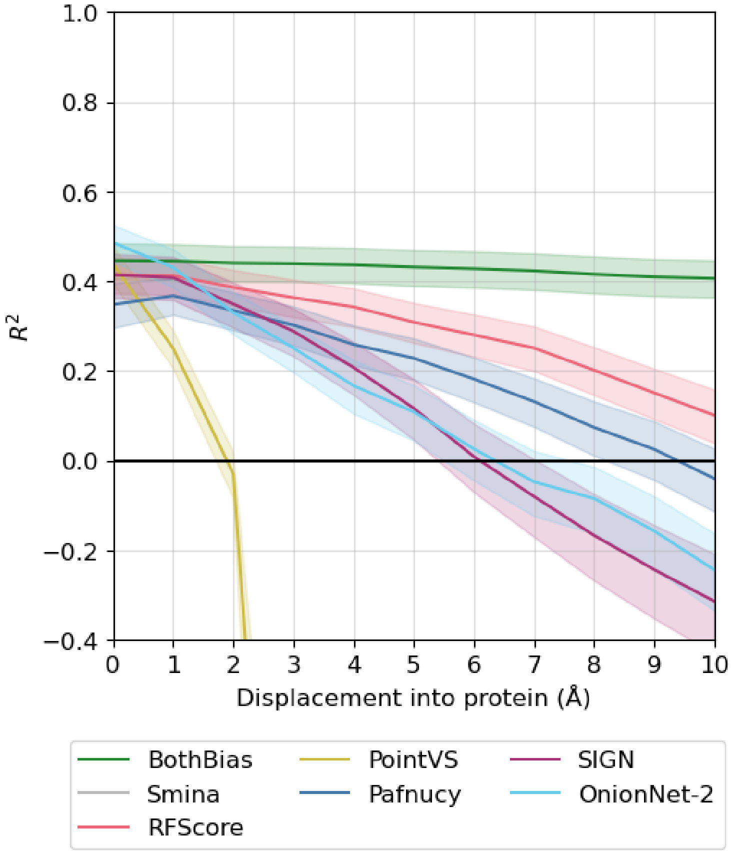

Figure 22: R^2 between predicted and true pK values for protein-ligand com-plexes for our baseline model (BothBias), a non-ML-based scoring function (Smina) and five commonly used MLBSFs (RFScore, PointVS, Pafnucy, SIGN and OnionNet-2), on progressively displaced ligands into the protein originally from 2019 Holdout crystal structures. Errors are the 95% confidence intervals from the bootstrapped R^2 (N=10000).

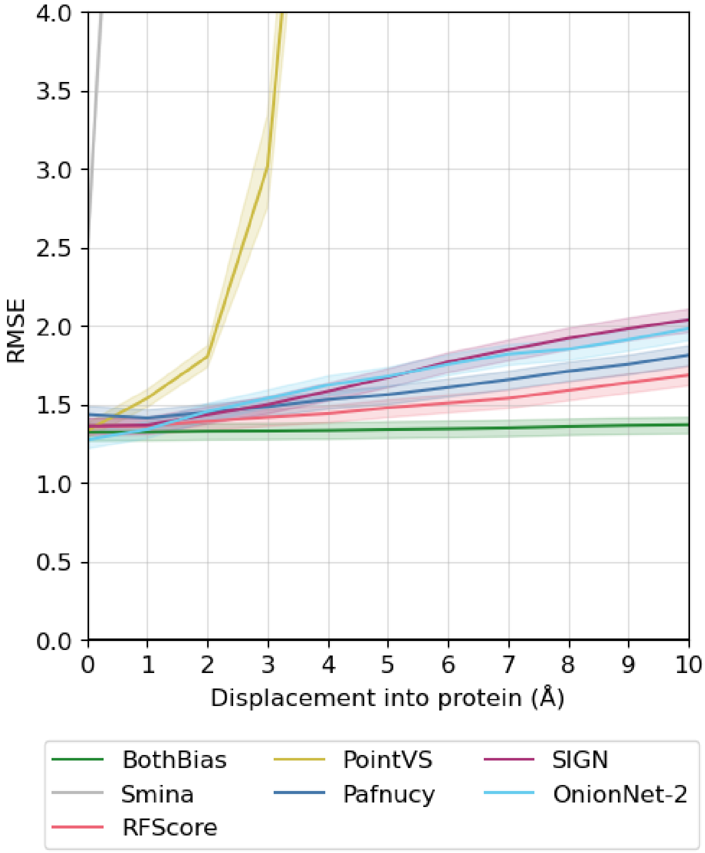

Figure 23: RMSE between predicted and true pK values for protein-ligand com-plexes for our baseline model (BothBias), a non-ML-based scoring function (Smina) and five commonly used MLBSFs (RFScore, PointVS, Pafnucy, SIGN and OnionNet-2), on progressively displaced ligands into the protein originally from 2019 Holdout crystal structures. Errors are the 95% confidence intervals from the bootstrapped RMSE (N=10000).

11 Accuracy of scoring functions and baseline models on progressively displaced ligands of 0 Ligand Bias

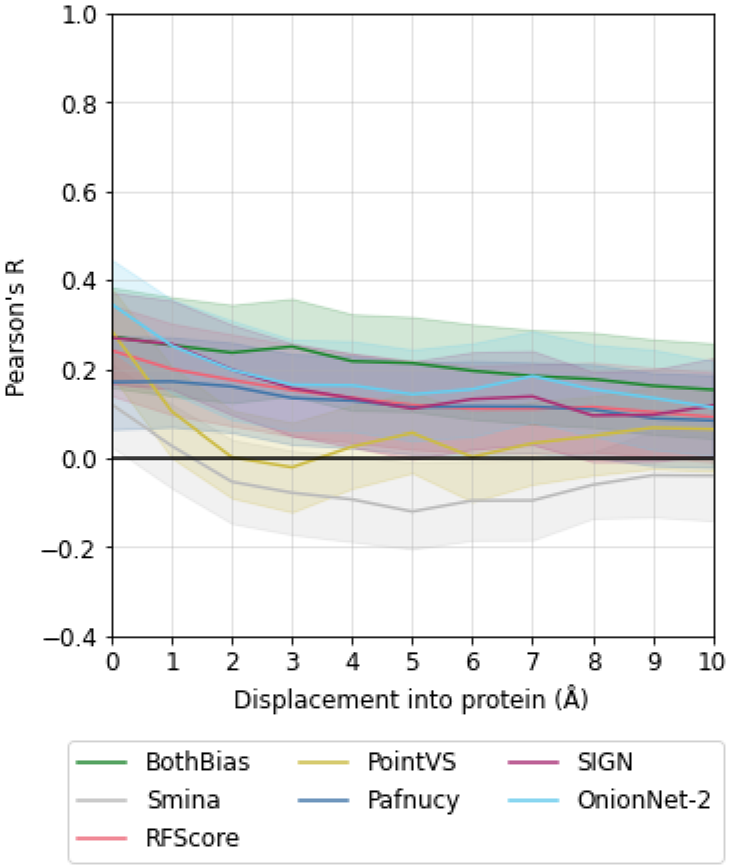

Figure 24: Pearson's R between predicted and true pK values for protein-ligand com- plexes for our baseline model (BothBias), a non-ML-based scoring function (Smina) and five commonly used MLBSFs (RFScore, PointVS, Pafnucy, SIGN and OnionNet-2), on progressively displaced ligands into the protein originally from 0 Ligand Bias crystal structures. Errors are the 95% confidence intervals from the bootstrapped Pearson's R (N=10000).

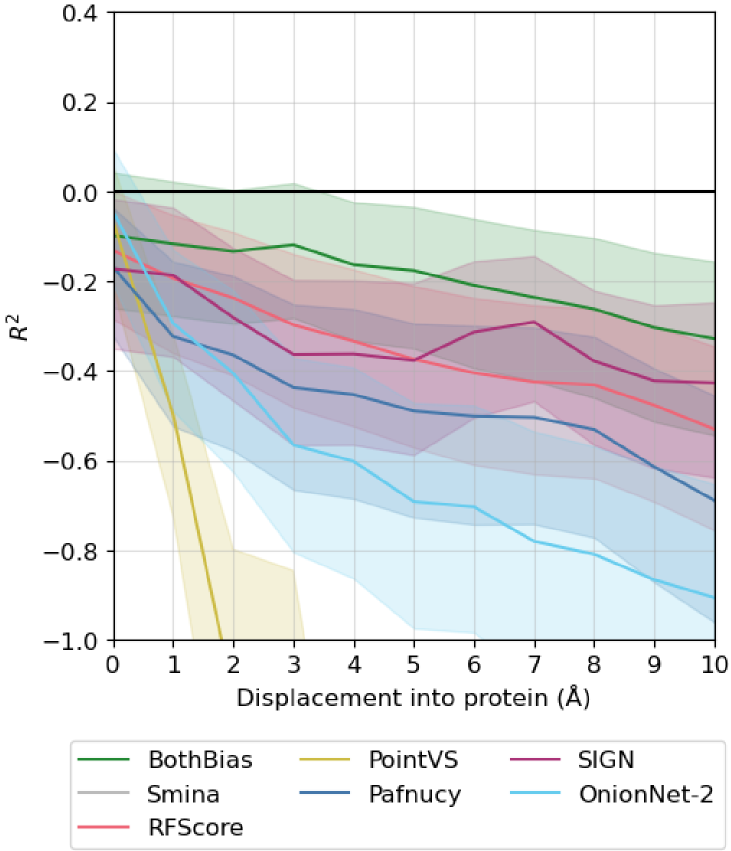

Figure 25: R^2 between predicted and true pK values for protein-ligand com-plexes for our baseline models (LigandBias, ProteinBias and BothBias), a non-ML-based scoring function (Smina) and five commonly used MLBSFs (RFScore, PointVS, Pafnucy, SIGN and OnionNet-2), on progressively displaced ligands into the protein originally from 0 Ligand Bias crystal structures. Errors are the 95% confidence intervals from the bootstrapped R^2 (N=10000).

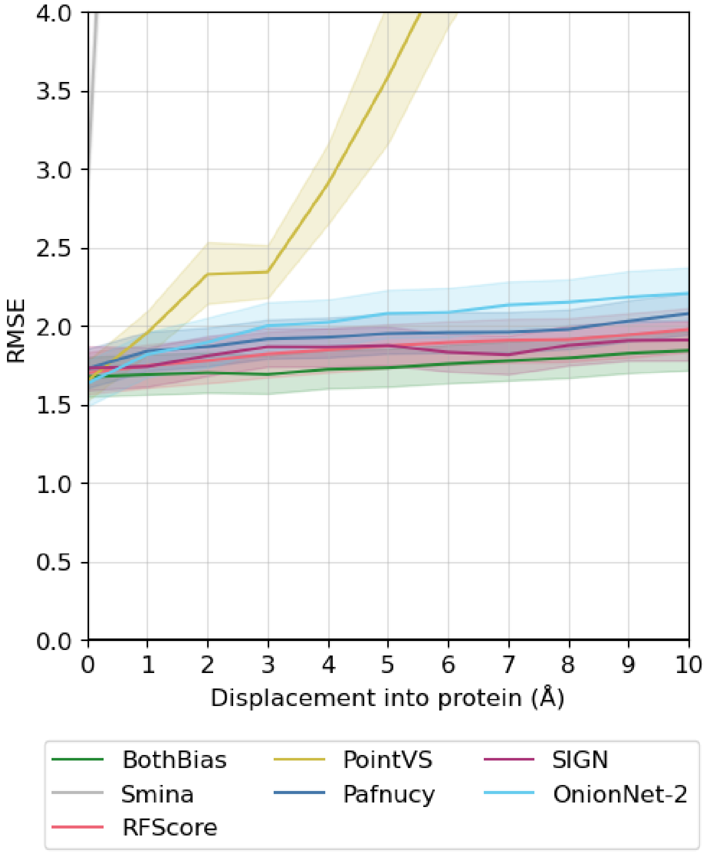

Figure 26: RMSE between predicted and true pK values for protein-ligand com-plexes for our baseline models (LigandBias, ProteinBias and BothBias), a non-ML-based scoring function (Smina) and five commonly used MLBSFs (RFScore, PointVS, Pafnucy, SIGN and OnionNet-2), on progressively displaced ligands into the protein originally from 0 Ligand Bias crystal structures. Errors are the 95% confidence intervals from the bootstrapped RMSE (N=10000).

References

Ballester, P. J. and Mitchell, J. B. (2010). A machine learning approach to predicting protein-ligand binding affinity with applications to molecular docking. *Bioinformatics*, **26**(9), 1169–1175.

Bickerton, G. R., Paolini, G. V., Besnard, J., Muresan, S., and Hopkins, A. L. (2012). Quantifying the chemical beauty of drugs. *Nature chemistry*, **4**(2), 90–98.

Diedrich, K., Krause, B., Berg, O., and Rarey, M. (2023). PoseEdit: Enhanced ligand binding mode communication by interactive 2D diagrams. *Journal of Computer-Aided Molecular Design*, **37**(10), 491–503.

Evans, R., O'neill, M., Pritzel, A., Antropova, N., Senior, A., Green, T., Žídek, A., Bates, R., Blackwell, S., Yim, J., Ronneberger, O., Bodenstein, S., Zielinski, M., Bridgland, A., Potapenko, A., Cowie, A., Tunyasuvunakool, K., Jain, R., Clancy, E., Kohli, P., Jumper, J., and Hassabis, D. (2022). Protein complex prediction with AlphaFold-Multimer.

Goddard, T. D., Huang, C. C., Meng, E. C., Pettersen, E. F., Couch, G. S., Morris, J. H., and Ferrin, T. E. (2018). UCSF ChimeraX: Meeting modern challenges in visualization and analysis. *Protein Science*, **27**(1), 14–25.

Jumper, J., Evans, R., Pritzel, A., Green, T., Figurnov, M., Ronneberger, O., Tunyasuvunakool, K., Bates, R., Žídek, A., Potapenko, A., Bridgland, A., Meyer, C., Kohl, S. A. A., Ballard, A. J., Cowie, A., Romera-Paredes, B., Nikolov, S., Jain, R., Adler, J., Back, T., Petersen, S., Reiman, D., Clancy, E., Zielinski, M., Steinegger, M., Pacholska, M., Berghammer, T., Bodenstein, S., Silver, D., Vinyals, O., Senior, A. W., Kavukcuoglu, K., Kohli, P., Hassabis, D., and Hassabis, D. 2021). Highly accurate protein structure prediction with AlphaFold. *Nature*, **596**, 583.

Li, S., Zhou, J., Xu, T., Huang, L., Wang, F., Xiong, H., Huang, W., Dou, D., and Xiong, H. (2021). Structure-aware Interactive Graph Neural Networks for the Prediction of Protein-Ligand Binding Affinity; Structure-aware Interactive Graph Neural Networks for the Prediction of Protein-Ligand Binding Affinity. *KDD*, **21**.

Mitchell, A. L., Almeida, A., Beracochea, M., Boland, M., Burgin, J., Cochrane, G., Crusoe, M. R., Kale, V., Potter, S. C., Richardson, L. J., Sakharova, E., Scheremetjew, M., Korobeynikov, A., Shlemov, A., Kunyavskaya, O., Lapidus, A., and Finn, R. D. (2019). MGnify: the microbiome analysis resource in 2020. *Nucleic Acids Research*, **48**(D1), D570–D578.

Pedregosa, F., Varoquaux, G., Gramfort, A., Michel, V., Thirion, B., Grisel, O., Blondel, M., Prettenhofer, P., Weiss, R., Dubourg, V., *et al.* (2011). Scikit-learn: Machine learning in Python. *Journal of machine learning research*, **12**(Oct), 2825–2830.

RCBS (2023). RCSB PDB: Programmatic Access - File Download Services - Sequence Clusters Data. Accessed on June 28, 2023.

Santos, G. B., Ganesan, A., and Emery, F. S. (2016). Oral administration of peptide-based drugs: beyond lipinski's rule. *ChemMedChem*, **11**(20), 2245–2251.

Scantlebury, J., Vost, L., Carbery, A., Hadfield, T. E., Turnbull, O. M., Brown, N., Chenthamarakshan, V., Das, P., Grosjean, H., von Delft, F., and Deane, C. M. (2023). A Step Towards Generalisability: Training a Machine Learning Scoring Function for Structure-Based Virtual Screening. *bioRxiv*, page 2022.10.28.511712.

Schrödinger, LLC (2015). The PyMOL molecular graphics system, version 1.8.

Stepniewska-Dziubinska, M. M., Zielenkiewicz, P., and Siedlecki, P. (2018). Development and evaluation of a deep learning model for protein–ligand binding affinity prediction. *Bioinformatics*, **34**, 3666.

Suzek, B. E., Huang, H., McGarvey, P., Mazumder, R., and Wu, C. H. (2007). UniRef: comprehensive and non-redundant UniProt reference clusters. *Bioinformatics*, **23**(10), 1282–1288.

Wang, Z., Zheng, L., Liu, Y., Qu, Y., Li, Y. Q., Zhao, M., Mu, Y., and Li, W. (2021). OnionNet-2: A Convolutional Neural Network Model for Predicting Protein-Ligand Binding Affinity Based on Residue-Atom Contacting Shells. *Frontiers in Chemistry*, **9**, 913.

Wójcikowski, M., Zielenkiewicz, P., and Siedlecki, P. (2015). Open Drug Discovery Toolkit (ODDT): a new open-source player in the drug discovery field. *Journal of cheminformatics*, **7**(1), 1–6.

Zhang, Y. and Skolnick, J. (2004). Scoring function for automated assessment of protein structure template quality. *Proteins: Structure, Function, and Bioinformatics*, **57**(4), 702–710.

Zhang, Y. and Skolnick, J. (2005). TM-align: a protein structure alignment algorithm based on the TM-score. *Nucleic Acids Research*, **33**(7), 2302–2309.
